# ITSN1 regulates SAM68 solubility through SH3 domain interactions with SAM68 proline-rich motifs

**DOI:** 10.1007/s00018-020-03610-y

**Published:** 2020-08-11

**Authors:** S. Pankivskyi, D. Pastré, E. Steiner, V. Joshi, A. Rynditch, L. Hamon

**Affiliations:** 1 SABNP, Univ Evry, INSERM U1204, Université Paris-Saclay, 91025 Evry, France; 2 Institute of Molecular Biology and Genetics, The National Academy of Sciences, 150 Zabolotnogo Street, Kyiv, 03680 Ukraine

**Keywords:** RNA-binding protein, Nuclear body, Intersectin, SAM68, SH3 domain, RNA, Proline-Rich Motif, Membraneless compartments

## Abstract

SAM68 is an mRNA-binding protein involved in mRNA processing in the nucleus that forms membraneless compartments called SAM68 Nuclear Bodies (SNBs). We found that intersectin 1 (ITSN1), a multidomain scaffold protein harboring five soluble SH3 domains, interacts with SAM68 proline-rich motifs (PRMs) surrounded by self-adhesive low complexity domains. While SAM68 is poorly soluble in vitro, the interaction of ITSN1 SH3 domains and mRNA with SAM68 enhances its solubility. In HeLa cells, the interaction between the first ITSN1 SH3 domain (SH3A) and P0, the N-terminal PRM of SAM68, induces the dissociation of SNBs. In addition, we reveal the ability of another SH3 domain (SH3D) of ITSN1 to bind to mRNAs. ITSN1 and mRNA may thus act in concert to promote SAM68 solubilization, consistent with the absence of mRNA in SNBs in cells. Together, these results support the notion of a specific chaperoning of PRM-rich SAM68 within nuclear ribonucleoprotein complexes by ITSN1 that may regulate the processing of a fraction of nuclear mRNAs, notably SAM68-controlled splicing events related to higher neuronal functions or cancer progression. This observation may also serve as a putative model of the interaction between other PRM-rich RBPs and signaling proteins harboring SH3 domains.

**Electronic supplementary material:**

The online version of this article (10.1007/s00018-020-03610-y) contains supplementary material, which is available to authorized users.

## Introduction

Membraneless compartments participate in the organization of cellular constituents [1–4] and initiate specific biological processes by concentrating constituents and possibly increasing processing rates [5–7], regulating the specific translocation or exclusion of biomolecules from a given compartment [8], and controlling compartment assembly and dissociation [9–11]. Many membraneless compartments have a nuclear localization and are gathered under the name of nuclear bodies (NBs), such as nucleoli, paraspeckles, and Cajal bodies [12, 13]. NBs are critical for the processing (splicing, transcription) and biogenesis of RNA (assembly of ribosomes) [13]. RNA-Binding Proteins (RBPs) and RNA are the major NB constituents [12]. Many RBPs involved in NB assembly harbor low complexity domains (LCD) characterized by an enrichment in a limited number of amino acids and an absence of 3D structure, which provide the necessary intermolecular multivalent interactions to keep the liquid-like behaviour of compartments [9, 14–16]. In addition, RNA serves as a crucible by directing many RBPs on their surface to promote LCD interactions [9, 16–19] and generates by itself weak RNA/RNA interactions that participate in compartment assembly [20].

However, while many nuclear RBPs have the capacity to self-aggregate and generate condensates, they have to remain soluble to sustain their function and what preserves the solubility of these proteins in the nucleus has been under focus recently [17, 21, 22]. An advanced hypothesis is the high content of RNA in the nucleus which prevents condensate formation by dispersing RBPs [17], even though RNA in excess may generate RNA base-pairings in the nucleus to promote droplet assembly [20, 23, 24]. In addition to RNA buffering, some nuclear proteins that remain to be identified may prevent an overall aggregation of RBPs containing LCD and act as chaperones to negatively regulate compartment formation. Proteins displaying SRC homology 3 domain (SH3) are good candidates to fulfill this task. Preferential interactions between SH3 domains and the proline-rich motifs (PRMs) which are common in cell regulatory systems [25], may regulate the formation of RBP condensates, dispersing them on many SH3-rich scaffolds [26] or by masking multivalent interactions between LCDs in RBPs harboring a combination of LCDs and PRMs [26]. Like for RNA, the buffering efficiency of RBPs containing PRMs is relying on the SH3 domains concentration and, consequently, on the number of the domains in protein [27]. Among the few proteins that have an elevated number of SH3 domain copies to efficiently disperse RBPs condensates is intersectin 1 (ITSN1). ITSN1 exists in two main isoforms, short or long, generated by alternative splicing and harbors five consecutive SH3 domains. ITSN1, which was first described as an endocytosis protein [28], is considered as a scaffold protein due to its involvement in various cytoplasmic processes like actin cytoskeleton rearrangements and cell signaling [29, 30]. Consistent with this view, ITSN1 has been associated with neuronal diseases [31, 32]. However, ITSN1 has been also found in the cell nucleus with unknown functions [33]. The identification of several RBPs as potential ITSN1 partners by previous high throughput screenings [34–36] led us to hypothesize that the SH3 domains of ITSN1 could be involved in RBP processing in the nucleus.

To explore the putative role of ITSN1 in controlling the spatial distribution of RBPs, we first determined whether the interactions between ITSN1 and RBPs harboring PRMs were relevant in a cellular context. The results indicate that, among the selected RBPs, SRC associated in mitosis of 68-kDa protein (SAM68) interacts significantly with ITSN1. SAM68 is a member of the STAR family, composed of proteins that are conserved through yeast, mammals and plants. SAM68 controls various aspects of RNA metabolism, including pre-mRNA splicing. Besides its central and conserved RNA-binding domain, SAM68 possesses flanking proline-rich motifs and RG-rich domains that are prone to self-attraction making SAM68 an insoluble protein in vitro [37–39]. In cells, SAM68 is mostly homogenously distributed in the nucleoplasm but also accumulates in small structures called SAM68 nuclear bodies (SNBs) [40]. Through a combination of biochemical analysis, nanoscopic observations by atomic force microscopy (AFM), and cellular investigations by fluorescence microscopy, we dissected the interaction of the SH3 domains of ITSN1 with the SAM68 PRMs. Additional biochemical analyses also showed that multivalent SH3-PRM interactions solubilize SAM68 aggregates in vitro and that mRNA potentiates the ITSN1-mediated SAM68 solubilization. In agreement with the latter point, ITSN1 interacts with mRNA through one of its SH3 domains, SH3D. Using NMR spectroscopy, specific nucleic acid-interacting residues that are not conserved in other ITSN1 SH3 domains were identified in SH3D. In HeLa cells, we then evidenced a negative correlation between ITSN1 expression or ITSN1 levels in the nucleus and the SAM68 enrichment in SNBs in which both mRNA and ITSN1 were not detected, thus corroborating the results obtained in vitro. At the light of the results presented in this study, we propose a mechanistic model which describes the intermolecular interaction between SH3, PRM domains and mRNA. This tripartite interaction participates in the control of the formation of liquid-like SAM68 condensates and provide an additional layer of complexity in mRNA splicing that is still poorly understood.

## Materials and methods

### Plasmid constructs

The preparation of expression constructs encoding proteins of interest fused to RFP and Microtubule-Binding Domain of Tau (MBD) was described in details previously [41]. In brief, cDNA of the full length SAM68 and cDNA encoding truncated form of ITSN1 (ITSN1_SH3_) containing five SH3 domains (residues 730–1220, Accession number NP_001001132.1) were amplified using primers containing the PacI and AscI restriction sites and inserted into the backbone entry plasmid RFP-MBD-pCR8/GW/TOPO previously digested with the corresponding restriction enzymes. The inserted cDNAs were verified by sequencing. Next, sequences encoding ITSN1_SH3_-RFP-MBD and SAM68-RFP-MBD were subcloned from pCR8/GW/TOPO plasmids into the Gateway^®^ pEF-Dest51 plasmid (Invitrogen™) using the LR recombination reactions (Invitrogen™) according to the manufacturer’s protocol. The preparation of plasmid pEF-Dest51-TDP43-RFP-MBD was described previously [42].

For the preparation of constructs expressing GFP-fused truncated forms of ITSN1, cDNAs encoding two EH domains (ITSN1_EH_, residues 1–316), EH-domains and CCR (ITSN1_EH-CCR_, residues 1–736), SH3 domains (ITSN1_SH3_, residues 730–1220), and CCR with SH3 domains (ITSN1_CCR-SH3_, residues 310–1220) were amplified by PCR and inserted into pEGFP-C1 vector (Clontech) using EcoRI and SalI restriction sites. Truncated form of ITSN1 lacking the first EH domain (ITSN1_ΔEH1_, residues 187–1220) was prepared by deleting EH1-coding sequence from the plasmid pEGFPC1-ITSN1s using BglII restriction enzyme with further self-ligation of the construct. Truncated form of ITSN1 lacking EH1 domain and the first SH3 domain (ITSN1_ΔEH1-ΔSH3A_) was prepared in two steps. First, EH1-encoding sequence was deleted from the plasmid pEGFPC1- ITSN1_EH-CCR_ using BglII restriction enzyme, and the plasmid was self-ligated. Second, the sequence encoding four last SH3 domains of ITSN1 (residues 811–1220) was amplified by PCR and inserted into the aforementioned construct using SalI and BamHI restriction sites. The plasmid encoding full-length ITSN1s fused to GFP was described previously [43].

For the preparation of constructs expressing GFP-fused truncated forms of SAM68, amplified cDNAs encoding N-terminal unstructured region with KH domain (SAM68_N-term-KH_, residues 1–282), KH domain (SAM68_KH_, residues 95–282), and KH domain with C-terminal unstructured region (SAM68KH-C-term, residues 95–443) were inserted into pEGFP-C1 vector. Truncated form of SAM68 lacking the first proline motif (SAM68_ΔP0_, residues 51–443) was inserted into the pEGFPC1 vector using BamHI/BglII and SalI restriction sites.

For the production of the plasmid encoding SAM68 fused to His tag, the coding sequence of full-length SAM68 was amplified from the plasmid GFP-SAM68 using PCR and inserted into the pET22b vector (Novagen). For the preparation of constructs encoding the truncated form of ITSN1 containing five SH3 domains (ITSN1_SH3_, residues 730–1220) fused to His or GST tag, the amplified coding sequence was inserted into plasmids pET22b (Novagen) and pGEX-4T1 (GE Healthcare), respectively. The constructs encoding GST-fused SH3 domains of ITSN1 and SRC kinase were described previously [44, 45]. For the production of the plasmid encoding ITSN1 SH3D domain (residues 1063–1150) fused to His tag, the corresponding sequence was amplified from the plasmid pEGFPC1-ITSN1s using PCR and inserted into the pET22b vector (Novagen).

Constructions encoding shRNAs specific to ITSN1 mRNA were prepared according to the RNAi Consortium (TRC) protocol [46]. The sequences of ITSN1-specific shRNAs were obtained from TRC Library (shRNA_ITSN1_-1 sense sequence: 5′-GAT ACT CAA TGA CCA ATT AAA-3′, shRNA_ITSN1_-2 sense sequence: 5′-CAC TAG CTG ACA TGA ATA AT-5′). The corresponding oligonucleotides pairs were annealed following incubation at 95 °C for 5 min and slowly cooling to room temperature overnight. The products of the annealing were inserted into pLKO.1 vector using AgeI and EcoRI restriction enzymes.

The inserted cDNAs and reading frames for all prepared plasmids were verified by sequencing.

Expression constructs encoding SAM68-GFP was a kind gift of Dr. D. J. Elliott [47], WBP11-GFP was received from Dr. M.Bollen [48] and LARP6-GFP was obtained from Dr. L. M. Schwartz [49].

### Cell culture

HEK-293 and HeLa cells were obtained from the American Type Culture Collections (ATCC) and were maintained at 37 °C and 5% CO_2_ in DMEM medium (Life Technologies) supplemented with 10% fetal bovine serum (Life Technologies), 100 U/ml penicillin and 100 μg/ml streptomycin. Transient transfections were performed using indicated plasmid DNA of appropriate concentrations and Lipofectamine 2000™ reagent (Invitrogen) according to the manufacturer’s protocol. Transfected cells were processed 24 h post transfection.

### shRNA transduction

ITSN1 knockdown in HeLa cells was performed using lentivirus particles produced in HEK-293 cells according to TRC protocols [46]. In brief, HEK-293 cells plated in 5 mL of media in a 6 cm tissues culture plate were transfected with a plasmid mix consisting of i) 1 µg empty pLKO.1 or shRNA-pLKO.1 plasmid, ii) 1 µg psPAX2 packaging plasmid, and iii) 0.5 µg pMD2.G envelope plasmid. The transfections were performed using Lipofectamin 2000 according to the manufacture’s protocol. Following the incubation at 37 °C, 5% CO_2_ for 15 h, transfection reagent was removed by changing the growth medium, whereas cells were incubated at the same conditions for 48 h. The media containing lentiviral particles was harvested, filtered through a 0.45 μm and stored at − 80 °C in single-use aliquots. For ITSN1 knockdown, 70% confluent HeLa cells were added 300 μl of lentivirus-containing media and were incubated for 24 h. Next, cells were replaced with the fresh media containing selective antibiotic puromycin (1 μg/ml) and incubated for the next 72 h. At that time point, HeLa cells were processed for Western blot analysis or immunofluorescent staining.

### Fluorescent microscopy

For fluorescent microscopy analysis and microtubule bench assay, HeLa cells were grown on 12 mm coverslips in 24- or 4-well plates. Cells were fixed with ice-cold methanol for 15 min at − 20 °C, washed once with PBS, and fixed with 4% paraformaldehyde in PBS for 30 min at 37 °C. The double methanol/PFA fixation was previously shown to be the most efficient to maintain the microtubule structures [41]. Following washes with PBS, samples were prepared for fluorescent microscopy imaging by mounting the slides with MOWIOL reagent (Sigma).

For immunofluorescent staining, following the methanol/PFA fixation, HeLa cells were blocked and permeabilized in 2% BSA in PBS containing 0.2% Triton-X100 for 15 min. Then, cells were incubated for 2 h at room temperature or overnight at +4 °C with rabbit anti-ITSN1 (1:500; described in [45]) or mouse anti-SAM68 (diluted 1:20; clone 7-1; Santa Cruz Biotechnology, Inc.) antibodies followed by 2 h incubation with anti-rabbit or anti-mouse IgG conjugated with Alexa Fluor™ 594 or Alexa Fluor™ 488 (Invitrogen). Next, cells were washed with PBS, stained with 1 µg/ml DAPI for 5 min and mounted using MOWIOL. In situ hybridization was performed to detect poly(A) mRNA in HeLa cells. For the purpose, cells were fixed in methanol and PFA as described above and were incubated in ice-cold 70% ethanol for 10 min at -20 °C and 1 M Tris HCl (pH 8.0) for 5 min. Next, samples were incubated with Cy2-conjugated poly(T) probes (Sigma) at 1 μg/μL in the hybridization buffer (0.005% BSA, 1 mg/mL yeast RNA, 10% dextran sulphate, 25% formamide in 2 × SSC) in a humidity chamber for 1 h at 37 °C. Following the hybridization, samples were washed twice with 4 × SSC, once with 2 × SSC and mounted with MOWIOL.

Proximity ligation assay was performed using Duolink^®^ PLA technology Kit (Sigma) according to the manufacturer’s recommendations. Briefly, cells grown on 12 mm coverslips were fixed with 4% PFA for 20 min, permeabilized using 0.1% Triton in PBS for 5 min and blocked using blocking solution for 30 min. Samples were incubated with anti-ITSN1 (diluted 1:500) and anti-SAM68 (diluted 1:20) antibodies in supplied buffer at +4 °C overnight. Next, samples were incubated with PLA^®^ probes (diluted 1:5 in supplemented buffer) for 60 min at +37 °C. Following washing with PBS, samples were added ligation mix (diluted 1:5 ligation stock and 1:40 ligase) and incubated for 30 min at 37 °C. After washing with PBS, the samples were incubated with amplification mix (diluted 1:50 amplification stock and 1:80 Polymerase) for 100 min at +37 °C and washed with PBS. Finally, samples were stained with DAPI and mounted in MOWIOL.

Fluorescence emission was detected using an oil immersed 63 ×/1.4 NA objective on an inverted microscope (Axiovert 220; Carl Zeiss 5 MicroImaging, Inc). The images were processed and analyzed in ImageJ 1.52a software.

In microtubule bench assay, to estimate the co-localization level between a bait protein fused to RFP-MBD and a putative prey protein fused to GFP, correlation analysis was performed (Supplementary Figure S1). In brief, images of the bait (red channel) and the prey (green channel) were merged into a single red–green image. A line of 100–150 pixels crossing a cell region containing apparent microtubules was build and fluorescent intensities profiles along the line were generated for each channel. The line profile for each channel was transformed into numerical values by obtaining a list of the fluorescence intensities of all pixels along the line. The acquired lists were used to calculate the correlation coefficient between fluorescent intensities from red and green channels along a single line. Microsoft Excel CORREL function providing the calculation of Pearson correlation coefficient was used to estimate the co-localization level between two proteins. At least twenty lines from three independent experiments were analyzed for each bait-prey pair.

For the estimation of the nucleus/cytoplasm fluorescence ratio of GFP-tagged ITSN1s, mean fluorescent intensity from a cell nucleus and cytoplasm were calculated. Following the adjustments of a threshold for blue (DAPI) and green (GFP) channels, the nuclear region and the entire cell were selected using ROI manager. Next, mean and total fluorescent intensities of GFP-ITSN1s from the selected areas were measured. The cytoplasm fluorescence was estimated as the difference between integral fluorescent intensities of the entire cell and the nucleus. Mean cytoplasm fluorescence was determined by dividing total cytoplasm fluorescence by cytoplasm area.

To obtain SAM68 nuclear bodies (SNBs) through the entire volume of a cell nucleus, SAM68 fluorescence emission was analyzed from different focal planes with the step of 500 nm for each field of view (Supplementary Figure S10A) and images used for the measuring of SNBs intensity were captured at the fluorescence level below the saturation. Z-stacking of series of images was performed using ImageJ 1.52a software. Mean SAM68 fluorescence and maximum SNBs fluorescence intensities were measured in a DAPI-defined nuclear region for each cell. In the case of the absence of visible SNBs, maximum SAM68 fluorescence intensity in the nuclear region was used for further calculations. Finally, the ratio between peak height (the difference between maximum SNB intensity and mean value) and mean SAM68 intensity was estimated and used in the statistical analysis.

### Recombinant protein production and purification

Recombinant proteins SAM68-His, ITSN1_SH3_-His, ITSN1_SH3D_-His, GST-ITSN1_SH3_, SRC_SH3_-GST, and GST were expressed in *E. coli* BL21 (DE3) and purified as described below. Briefly, *E. coli* cells were transformed with pET22b-SAM68 plasmid, induced at OD600 0.8 by 1 mM IPTG and grown at 20 °C overnight. Cells were lysed by sonication in the buffer containing 20 mM Tris–HCl (pH 7.4), 1 M NaCl, 8 M urea, 1% Triton-X100, 10 mM imidazole, 1.5 mM β-mercaptoethanol, 1 mM phenylmethylsulfonylfluoride (PMSF) (Sigma), and protease inhibitors cocktail (Roche). SAM68-His was purified using Ni^2+^-NTA-agarose (Qiagen) following the manufacturer’s recommendations. Protein-containing fractions eluted with 250 mM imidazole were dialyzed overnight against 20 mM Tris–HCl buffer (pH 7.4), containing 150 mM NaCl and 8 M urea.

For the production of ITSN1_SH3_-His and ^15^N- or ^15^N/^13^C-labeled ITSN1_SH3D_-His, *E. coli* cells were transformed with the corresponding plasmids, incubated in 2YT medium (unlabeled proteins) or minimal medium M9 supplemented with ^15^N or ^15^N/^13^C (labeled proteins), induced at OD600 0.8 using 1 mM IPTG and grown at 30° for 4 h. Cells were lysed by sonication in the buffer containing 20 mM Tris–HCl (pH 7.4), 500 mM NaCl, 1% Triton-X100, 10 mM imidazole, 1.5 mM β-mercaptoethanol, 1 mM phenylmethylsulfonylfluoride (PMSF), and protease inhibitors cocktail (Roche). His-tagged ITSN1_SH3_ and ITSN1_SH3D_ were purified using Ni^2+^-NTA-agarose (Qiagen). Eluted ITSN1_SH3_-His was dialyzed overnight against buffer containing 20 mM Tris–HCl (pH 7.4) and 150 mM NaCl, whereas ^15^N- and ^15^N/^13^C-labeled ITSN1_SH3D_-His was dialyzed against 12 mM potassium phosphate buffer, 25 mM KCl and 1 mM tris(2-carboxyethyl)phosphine (TCEP) (Sigma).

For the production of recombinant proteins GST-fused SH3 domains and GST, *E. coli* cells were transformed with the corresponding plasmids, induced at OD600 0.8 by 1 mM IPTG, and grown at 37 °C for 4 h. Cells were lysed by sonication in the buffer containing 20 mM Tris–HCl (pH 7.4), 500 mM NaCl, 10% glycerol, 1% Triton-X100, 1.5 mM β-mercaptoethanol, 1 mM phenylmethylsulfonylfluoride (PMSF) (Sigma), and protease inhibitors cocktail (Roche). GST-tagged proteins and GST alone were purified from cell lysates using glutathione–Sepharose 4B (GE Healthcare) according to the manufacturer’s recommendations. Protein-containing fractions eluted with 20 mM glutathione were dialyzed overnight against buffer containing 20 mM Tris–HCl buffer (pH 7.4) and 150 mM NaCl.

All dialyzed protein samples were concentrated using Spin-X^®^ UF Concentrators (Corning), supplemented with 10% glycerol (except ^15^N- and ^15^N/^13^C-labeled ITSN1_SH3D_-His), snap frozen in liquid nitrogen, and stored at -80 °C. Recombinant human TDP-43 was purchased from Abcam (ab156345).

### In vitro protein binding assay

For the analysis of proteins binding in vitro, purified recombinant proteins SAM68-His, TDP43-His, GST-ITSN1_SH3_ and GST-SRC_SH3_ were used. Briefly, 1 µg of purified SAM68-His or 1 µg of TDP43-His was incubated with 40 µl of pre-washed 50% Ni–NTA-agarose (Qiagen) in 500 µl of binding buffer (25 mM Tris–HCl (pH 7.4), 150 mM NaCl, 10% glycerol, and 1% Triton-X100) containing 8 M urea at 4 °C for 1 h with thorough mixing. Following washing with the buffer containing 8 M urea, Ni–NTA beads with immobilized proteins were washed once with binding buffer without urea. Next, SAM68- and TDP-43-containing beads were incubated with 1 µg of GST-SRC_SH3_, GST-ITSN1_SH3_ or GST in 500 µl of binding buffer at 4 °C for 1 h with thorough mixing. Following the washing procedures, bead-associated proteins were eluted in Laemmli buffer containing 250 mM imidazole, resolved by PAGE-SDS, and stained with Coomassie dye.

For GST-pull-down assay, 10 µg of purified GST, GST-fused SH3 domains of ITSN1 and SRC were incubated with 30 µl of pre-washed 50% glutathione–Sepharose 4B in 500 µl of binding buffer at 4 °C for 1 h. Following washing steps, the beads were incubated with HEK-293 cells lysates prepared in the extraction buffer (25 mM Tris–HCl pH 7.4, 150 mM NaCl, 0.5% Triton X-100, 1 mM EDTA, and protease inhibitors cocktail (Roche) at 4 °C for 1 h. Beads-associated proteins were eluted in Laemmli buffer, resolved by PAGE-SDS, and stained with Coomassie dye.

### Protein–mRNA complexes analysis

#### In vitro RNA transcription

RNA for the analysis of protein–RNA complexes was produced by in vitro transcription procedure. For this purpose, linearized plasmid pSP72-2Luc, containing separated by a polylinker two full-length cDNAs encoding *Renilla reinformis* and *Photinus pyralis* luciferases, was used as a template for synthesis 2Luc mRNA (∼ 3000 nt). Transcription in vitro was performed by a HiScribe T7 High Yield RNA Synthesis Kit (NEB) according to the manufacturer’s protocol. Synthesized RNA was purified using phenol/chloroform extraction.

#### Electrophoretic RNA mobility shift assay

For gel mobility shift assay, indicated amounts of recombinant proteins SAM68-His (2 pmol, 4 pmol, 8 pmol, 16 pmol, 32 pmol, and 64 pmol), ITSN1_SH3_-His (5 pmol, 10 pmol, 20 pmol, 40 pmol, 80 pmol, and 160 pmol) or GST-fused SH3 domains (~ 80 pmol) were incubated with 0.4 pmol of 2Luc mRNA in 20 mM Tris–HCl (pH 7.4), 50 mM mM KCl, and 1 mM MgCl at room temperature for 5 min. Protein–RNA complexes were separated in 0.7% agarose gel in 0.5 × TBE buffer at room temperature at 25 V for 1 h and stained with 0.5 µg/ml ethidium bromide.

#### Atomic force microscopy

The analysis of protein/RNA complexes using AFM technique was performed as described previously [50]. In brief, SAM68-His (25 nM) or ITSN1_SH3_-His (20 nM) was incubated with 0.3 nM 2Luc mRNA in AFM deposition buffer (15 mM KCl, 10 mM Tris–HCl (pH 7.5), 3 mM Putrescine Pu^2+^) at 37 °C for 5 min. Next, a 10 μl droplet was deposited on the surface of freshly cleaved mica at room temperature for 20 s. The mica surface was rinsed with 0.02% uranyl acetate solution and dried with a filter paper. AFM images recorded in air were performed on the Nanoscope V Multimode 8 (Bruker, Santa Barbara, CA) in PeakForce Tapping (PFT) mode enabling continuous force–distance curves recording using Scanasyst-Air probes (Bruker). Images were captured at 2048 × 2048 pixels at a line rate of 1.5 Hz. The Nanoscope Analysis software (version 1.50) was used to analyze dimensions of protein/RNA particle including the maximum height and the surface area of the particle.

#### Sedimentation assay

Recombinant SAM68-His (20 pmol) in the absence or presence of 2Luc mRNA (0.4 pmol) was incubated in a binding buffer containing 20 mM Tris–HCl (pH 7.4), 50 mM mM KCl, and 1 mM MgCl for 5 min at room temperature to favor the formation of SAM68-mRNA complexes. Next, GST-ITSN1_SH3_ (5 pmol, 10 pmol, 20 pmol, 40 pmol) or GST (45 pmol) was added to the SAM68-mRNA mixture and incubated in a total volume of 15 µl for 10 min at room temperature. Each sample was centrifuged at 1000 rpm for 1 min at +4 °C to pellet SAM68 aggregates. Supernatants were collected, whereas pellets were resuspended in 15 µl of binding buffer. Proteins present in pellet and supernatant samples were analyzed by 10% SDS-PAGE and Coomassie staining. The intensity of signals corresponding to protein bands was evaluated using ImageJ software.

#### Western blot

Protein samples in Laemmli buffer were resolved by SDS-PAGE and transferred to nitrocellulose membranes. The membranes were blocked with 5% non-fat milk in TBS containing 0.1% Tween 20 for 1 h, and incubated with mouse α-GFP (diluted 1:1000; clones 7.1 and 13.1; Roche), rabbit α-ITSN1 (diluted 1:20,000; described in [45]), or mouse α-GAPDH (diluted 1:20,000; clone 71.1; Sigma) antibodies for 1 h. Following washing, membranes were incubated with appropriate IRDye 700DX- or 800CW-labeled secondary antibodies and analyzed using Odyssey^®^ Infrared Imaging System (LI-COR, Inc.).

### NMR analysis

NMR experiments were conducted at 298 K on a Bruker AVIII HD 600 MHz spectrometer equipped with a triple-resonance cryoprobe and were processed with Topspin 3.5 package. ^1^H and ^15^N backbone chemical shifts of ITSN1 SH3D domain were assigned through the acquisition of standard 2D ^1^H-^15^N HSQC, 3D HNCA, 3D HNCACB, 3D HN(CO)CA, 3D HN(CO)CACB and 3D NOESY ^1^H-^15^N HSQC performed on a 300 μM [U-^15^N, U-^13^C] sample. Spectra were analyzed using CCPNmr Analysis 2.4.1 software [51]. To probe interactions with DNA, SOFAST-HMQC experiments [52] were acquired on samples containing 300 μM ^15^N-labeled SH3D domain free and in complex with CA_6_ or T_12_ ssDNA (Eurofins) at a 1:1.2 molar ratio. The number of dummy scans and scans was, respectively, set to 16 and 32. Shaped pulse length and power were calculated by considering an amide ^1^H bandwidth of 4.5 ppm and a chemical shift offset of 8.5 ppm. Data were recorded with 2048 points along the direct dimension, 128 t_1_ increments and a relaxation delay of 0.2 s corresponding to an experimental time of 23 min for each spectrum. For all experiments, 2,2- Dimethyl-2-silapentane-5-sulfonic acid was used as an external reference in pure D_2_O for chemical shift referencing.

### Statistics analysis

The analysis of the statistical significance and correlation analysis were performed in GraphPad Prism 6 software using unpaired two-tailed *t* test.

## Results

### The SH3 domains of ITSN1 interact with SAM68, an RNA-Binding Protein containing multiple PRMs

**Fig. 1:**
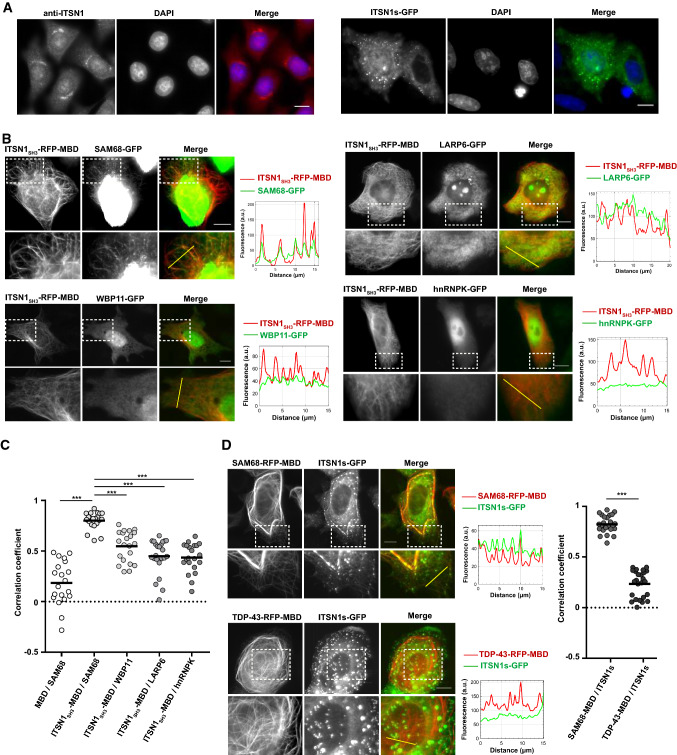
Scaffold protein ITSN1 localizes in HeLa cells nuclei and interacts with RBP SAM68 in the microtubule bench assay. **a**, left panel. Cellular distribution of endogenous ITSN1 in HeLa cells. ITSN1 was detected using anti-ITSN1 antibodies and Alexa Fluor 594-conjugated secondary antibodies. Nucleus was visualized using DAPI staining. Scale bar: 15 µm. **a**, right panel. Cellular distribution of the overexpressed ITSN1 short isoform (ITSN1s) in HeLa cells. HeLa cells were transfected with the plasmid encoding GFP-fused ITSN1s. Cells were fixed 24 h post-transfection. Nucleus was visualized using DAPI staining. Scale bar: 15 µm. **b** The results of the microtubule bench assay performed to identify the interaction between ITSN1 and selected RBPs. HeLa cells were co-transfected with the construction encoding ITSN1 fragment containing SH3 domains (ITSN1_SH3_) fused to RFP-MBD and the plasmid expressing one of the four tested RBPs (SAM68, WBP11, LARP6 or hnRNPK) fused to GFP. Scale bar: 15 µm. The line profile representing the fluorescence intensity from two channels is shown next to the respective microphotograph. **c** Scatter plot representing the co-localization level of MBD-fused ITSN1_SH3_ with one of the four tested RBPs and MBD with SAM68 as a control. Each data point represents a correlation coefficient between fluorescence intensities from red and green channels along the line crossing microtubules. The plot shows the data from three independent experiments. Lines show mean values. ***p < 0.0005, two-tailed *t* test. **d** HeLa cells were co-transfected with the constructions encoding SAM68 (upper panel) or TDP43 (lower panel) fused to RFP-MBD and the plasmid expressing full-length ITSN1s fused to GFP. TDP43 was used as a negative control as it binds RNA but it lacks potential ITSN1-interacting motifs. The line profile representing the fluorescence intensity from two channels is shown next to the respective micrograph. Scale bar: 15 µm. The scatter plot represents the co-localization level of MBD-fused SAM68 or TDP-43 with the full-length ITSN1s. Each data point represents a correlation coefficient between fluorescence intensities from red and green channels along a line crossing microtubules. The plot shows the data from three independent experiments. Lines show mean values. ***p < 0.0005, two-tailed *t* test

ITSN1 is a cytoplasmic scaffold protein that has also been identified in the cell nucleus [33]. To further clarify the nuclear location of ITSN1, we visualized the spatial distribution of endogenous ITSN1 in HeLa cells using specific anti-ITSN1 antibodies. A significant fraction of ITSN1 was detected in both perinuclear membranes and inside the nucleus. Ectopic GFP-ITSN1 expressed in HeLa cells was found in the cytoplasm but also, to a lesser extent than endogenous ITSN1, in the nucleus (Fig. 1a). Previous analyses of the Intersectin interactome have revealed few RBPs as putative ITSN1 partners among which four of them harbored multiples PRMs, SAM68 [34], WBP11 [36], LARP6 and hnRNPK [35] (Table 1). As the presence of the PRM ligands may generate preferential interactions with SH3 domains, we decided to screen the relevance of the putative interactions of ITSN1 with SAM68, WBP11, LARP6 and hnRNPK in HeLa cells using a recently developed technology called « microtubule bench » [41, 42]. The microtubule bench makes use, on the one hand, of a bait protein fused to the microtubule-binding domain of Tau (MBD) and a fluorescent label and, on the other hand, of a prey protein fused to a different fluorescent label that are expressed in mammalian cells. Due to its MBD, the bait protein is directed onto microtubules in cells. An interaction between bait and prey is then measured by scoring the co-localization level between bait and prey proteins along the microtubule network from fluorescence images (Supplementary Figure S1). To identify putative ITSN1 partners, HeLa cells were thus co-transfected with constructions encoding for the five SH3 domains of ITSN1 (ITSN1_SH3_) used as bait, and different RBPs, used as preys (Table 1, Fig. 1b). The spatial correlation coefficient between prey and bait fluorescence on microtubules then enables to score their interaction [41]. A value close to 1 denotes a strong co-localization between bait and prey proteins. The measured values of the correlation coefficients between ITSN1_SH3_ and different RBPs revealed that SAM68 has the highest interaction score with ITSN1_SH3_ domains compared to other RBPs containing multiples PRMs (Fig. 1c). As control experiments, no interaction could be detected between an empty bait protein (microtubule-binding domain fused to RFP) and SAM68 (Supplementary Figure S2). Given the strength of the interaction between ITSN1_SH3_ with SAM68 compared to LARP6, WBP11 and hnRNPK (Fig. 1c), we focused the rest of this study on the SAM68-ITSN1 interaction.

**Table 1:** Schematic representation of the domain structure of ITSN1s and RNA-binding proteins used in the microtubule bench assay to reveal the interaction with ITSN1

Protein name	Domain structure	Experimental evidence	References
ITSN1s			
Putative ITSN1 partners			
SAM68		Phage display	Asbach et al. [34]
WBP11		Two-hybrid screening	Thalappilly et al. [36]
LARP6		Two-hybrid screening*	Wong et al. [35]
hnRNPK		Two-hybrid screening*	Wong et al. [35]

The initial high throughput studies that identified the putative interaction are indicated

RG, Arginine–Glycine motif; P, PxxP motif; *, identified for ITSN2

### ITSN1 interacts with SAM68 in cells and in vitro through the SH3 domains

**Fig. 2:**
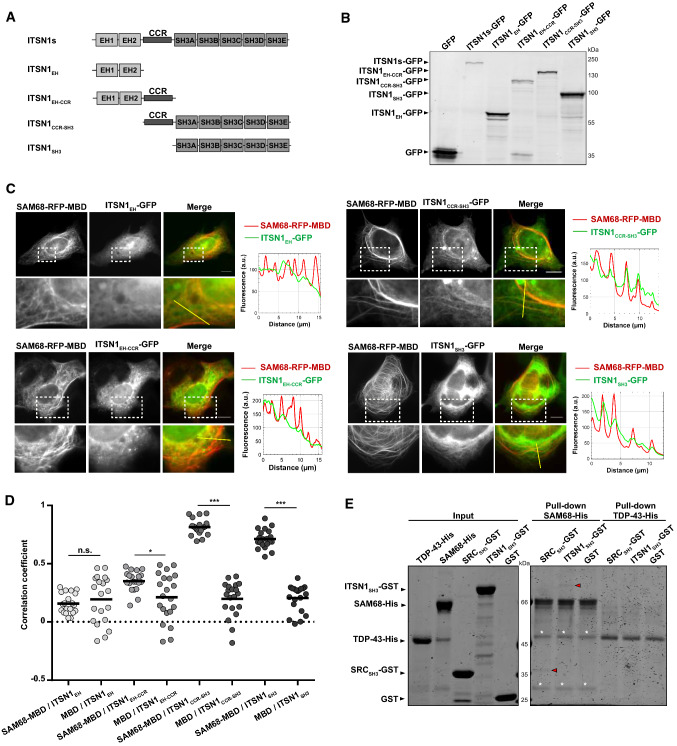
ITSN1 SH3 domains mediate direct binding to SAM68 in cellular context and in vitro. **a** Schematic representation of the domain structures of ITSN1s truncated forms used in the microtubule bench assay analysis to reveal the interaction between ITSN1s and SAM68. **b** Western blot analysis of the total lysates of HEK cells transfected with plasmids encoding indicated GFP-fused truncated forms of ITSN1s. The proteins were detected with anti-GFP antibodies. **c** The results of the microtubule bench assay confirming the interaction between SH3-containing truncated forms of ITSN1s and SAM68. HeLa cells were co-transfected with the construction encoding SAM68 fused to RFP-MBD and the plasmid expressing one of the truncated forms of ITSN1s fused to GFP. The line profile representing the fluorescence intensity from two channels is shown next to the respective micrograph. Scale bar: 15 µm. **d** Scatter plot representing the co-localization level between RFP-MBD-fused SAM68 or RFP-MBD alone (Supplementary Figure S6A) and truncated forms of ITSN1s. Each data point represents a correlation coefficient between fluorescence intensities from red and green channels along the line crossing microtubules. The plot shows the data from three independent experiments. Lines show mean values. *p < 0.05, ***p < 0.0005, *n.s.* not significant, two-tailed *t* test. **e** Pull-down assay confirming the direct binding of ITSN_SH3_ to SAM68 in vitro. TDP43 was used as a negative control, whereas SH3 domain of SRC kinase (SRC_SH3_) known to interact with SAM68 was used as a positive control. Proteins were visualized using Coomassie staining. Red arrows indicate GST-SRC_SH3_ and GST-ITSN1_SH3_ precipitated by SAM68-His. White asterisks indicate nonspecific products obtained during SAM68 purification

Endogenous SAM68 has a nuclear location and is not found on microtubules under physiological condition (Supplementary Figure S3). The observed interaction of SAM68 with ITSN1_SH3 _brought onto microtubules may, therefore, result from a significant affinity of SAM68 for ITSN1_SH3_ (Fig. 1b). To further probe the relevance of the interaction between SAM68 and ITSN1, SAM68 was in turn, used as a bait and brought onto microtubules in HeLa cells. In addition, we used full-length ITSN1 (of the short isoform, ITSN1s) instead of ITSN1_SH3_ as the prey protein to decipher whether other ITSN1 domains may not hinder the interaction with SAM68. A co-localization of ITSN1 with SAM68 is clearly evidenced on micrographs (Fig. 1d) but not when TDP-43, a RBP harboring a LCD but no PRM, was used as a negative control bait. In situ hybridization assays using a fluorescent poly(T) probes revealed that SAM68 directed onto microtubules has preserved its ability to bind to poly(A) mRNA (Supplementary Figure S4), which was also reported previously for TDP-43 [42]. As TDP-43 does not colocalize with ITSN1 on microtubules (Fig. 1d), the presence of mRNA on microtubules cannot account for the presence of ITSN1 on microtubules. When RBPs without PRM, like TDP-43, G3BP1, YB1 or FUS, were used as bait, no interaction with ITSN1_SH3_ was detected and, consistently, the correlation coefficient was close to 0 (Supplementary Figure S5 and Fig. 1d). The results presented so far indicate that the co-localization of ITSN1 and SAM68 can be mostly based on the affinity between the ITSN1 SH3 domains and SAM68 PRMs. To ascertain this working hypothesis, we generated constructs encoding ITSN1 fragments that contain different domains fused to GFP (Fig. 2a, b). Co-localizations between ITSN1 fragments and SAM68 were probed in HeLa cells expressing SAM68 as bait and ITSN1 fragments as preys (Fig. 2c). Control experiments with an empty bait protein revealed no ITSN1 presence along the microtubule network (Supplementary Figure S6A). Only ITSN1 fragments containing the SH3 domains showed a similar co-localization score with SAM68 (Fig. 2d) compared to full-length ITSN1 (Fig. 1d).

To further probe the relevance of the interaction of ITSN1 and SAM68 with alternative approaches to the microtubule bench, we analyzed the spatial distribution of endogenous ITSN1 and SAM68 via proximity ligation assays (PLA) performed according to the *Duolink*^*®*^ in situ *fluorescence* protocol and we confirmed the spatial proximity between ITSN1 and SAM68 in HeLa cells (Supplementary Figure S7A). To decipher whether ITSN1 interacts directly or indirectly with SAM68, in vitro pull-down assays analysis experiments were performed with recombinant proteins, SAM68-His, GST-ITSN1_SH3_, GST-SRC_SH3_ (SH3 domain of SRC kinase fused to GST) and TDP-43-His that were produced and purified in *E. coli*. GST-SRC_SH3_ was used as a positive control, since SAM68 is a well-known partner of SRC tyrosine kinase family [53, 54], while TDP43 is a negative control (Fig. 1d). Pull-down results demonstrated that SAM68 precipitated both the SH3 domains of SRC and ITSN1 but not TDP-43 and GST evidencing a direct interaction between SAM68 and SH3 domains (Fig. 2e). The interaction was also revealed by the saturation of SAM68 with ITSN1_SH3_ under the conditions of increased ITSN1_SH3_ amounts (Supplementary Figure S6B).

### ITSN1 modulates SAM68 solubility in vitro

**Fig. 3:**
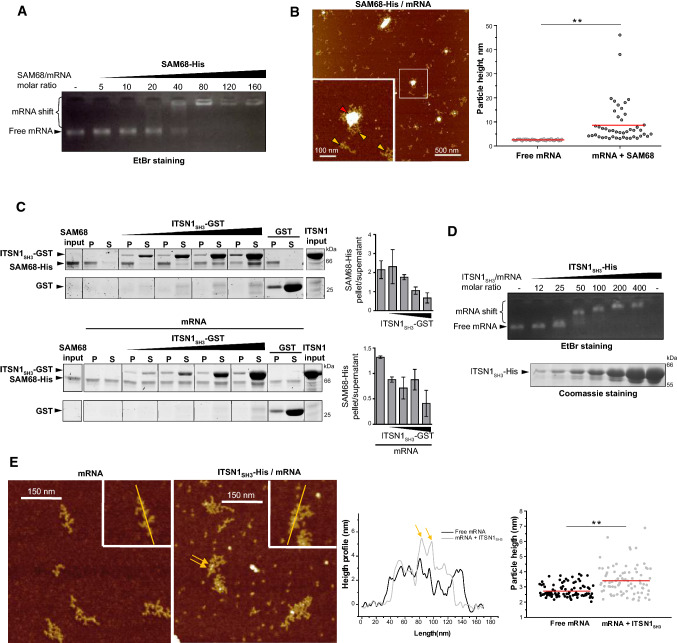
ITSN1 SH3 domains induce the dissociation of SAM68 aggregates and bind RNA in vitro. **a** RNA mobility shift assay demonstrating the formation of SAM68 aggregates. Recombinant SAM68-His was incubated with 0.4 pmol of 2Luc mRNA and resolved in agarose gel. **b** Atomic force microscopy images revealing the formation of SAM68-mRNA aggregates. Recombinant SAM68-His (25 nM) was incubated with 0.3 nM 2Luc mRNA in deposition buffer. Red arrow indicates SAM68 aggregate, while yellow arrows point mRNAs. Scatter plot represents the height of free mRNA molecules and mRNA/SAM68 complexes adsorbed on the same area. Z scale 7 nm. *n* = 100 molecules analyzed. Red lines show mean values. **p < 0.01, two-tailed *t* test. **c** Sedimentation assay results demonstrating that ITSN1_SH3_ induces the dissociation of SAM68 aggregates in vitro. SAM68-His (20 pmol) was incubated with GST-ITSN1 in the absence (upper panel) or presence (lower panel) of 2Luc mRNA (0.4 pmol). Following low speed centrifugation, proteins from pellet and supernatant fractions were resolved in SDS-PAGE and identified with Coomassie staining. The ratios between pellet and supernatant quantities of SAM68 were calculated from two independent experiments using ImageJ software and are shown as bars (mean ± SD). The experiment duplicate used for the calculation is shown in supplementary Figure S7B. **d** RNA mobility shift assay revealing the direct interaction between RNA and ITSN1_SH3_. Recombinant protein ITSN1_SH3_-His was incubated with 0.4 pmol of 2Luc mRNA and resolved in agarose gel (upper panel) or SDS-PAGE and stained with Coomassie (lower panel). **e** Atomic force microscopy images confirming the formation of ITSN1_SH3_-mRNA complexes. On the left panel, free 2Luc mRNA (0.3 nM) and on the right panel recombinant ITSN1_SH3_-His (20 nM) was incubated with 0.3 nM 2Luc mRNA in deposition buffer. Arrows point the presence of ITSN1_SH3_ interacting on mRNA. Z scale 7 nm. Height profiles along the yellow lines of the complex and free mRNA are compared. Scatter plot represents the height of mRNA in the absence or presence of ITSN1_SH3_. **p < 0.01, two-tailed *t* test

SAM68 has both structured and unstructured domains and displays a strong tendency for homodimerization and multimerization [37–39]. Consistently, SAM68 should be kept in high urea content after its purification to avoid its aggregation during storage. To probe the interaction of SAM68 with mRNA, we purified luciferase mRNA considered here as a long nonspecific mRNA control. SAM68 was then diluted in the presence of mRNA to prevent massive SAM68 aggregation and gel mobility shift assays revealed the binding of SAM68 to mRNA. We noticed the presence of two bands, one corresponding to free mRNA, the second to SAM68/mRNA complexes in the well (Fig. 3a). Most probably due to SAM68 self-attraction, the mixing of SAM68 with mRNA results in the formation of large SAM68/mRNA assemblies that remained stuck in the wells for a protein/RNA molar ratio above 40 (Fig. 3a). In addition the coexistence of free mRNA with large SAM68/mRNA assemblies reflects a cooperative binding of SAM68 to mRNA. Using the same SAM68/mRNA ratio, AFM imaging confirms the coexistence of free mRNA and large SAM68/mRNA granules (Fig. 3b), which is typical of a cooperative binding [55].

To test whether ITSN1 could regulate SAM68 self-assembly and large SAM68/mRNA assemblies, we performed sedimentation assays in the presence of mRNA or without it and an increasing amount of ITSN1_SH3_ (Fig. 3c, Supplementary Figure S7B). Short incubation time of SAM68 with ITSN1_SH3_ improves SAM68 solubility as increasing quantity of ITSN1_SH3_ progressively reduces the proportion of SAM68 in the pellet (Fig. 3c, upper panel). The interaction between ITSN1 and SAM68 may, therefore, prevent intramolecular interaction in SAM68 that are responsible for SAM68 aggregation. In the presence of mRNA, the proportion of SAM68 in the pellet is also decreased (Fig. 3c, lower panel). An increased RBPs solubility at elevated RNA concentrations has already been reported for RBPs such as FUS [50] which shares with SAM68 the ability to form large assemblies. Finally, simultaneous incubation of SAM68/mRNA complexes with ITSN1_SH3_ improves SAM68 solubility (Fig. 3c, lower panel). Moreover, mRNA and ITSN1 have a synergistic effect as a significant SAM68 solubility was obtained at lower concentration of ITSN1 when RNA is present. This result indicates that mRNA and ITSN1 may act in concert to prevent SAM68 intramolecular interactions. ITSN1 may interact with SAM68 PRMs, while mRNA binds to conserved KH domain of SAM68. Both can thus possibly bind to SAM68 at the same time. In addition, mRNA can be used as a crucible to promote the interaction between SAM68 and ITSN1. To probe this hypothesis, we analyzed the putative interaction between ITSN1 and mRNA. A direct interaction between ITSN1 SH3 domains and mRNA was detected both in gel shift assay (Fig. 3d) and by AFM imaging (Fig. 3e).

### Structural insights into the interactions between ITSN1, SAM68 and mRNA

**Fig. 4:**
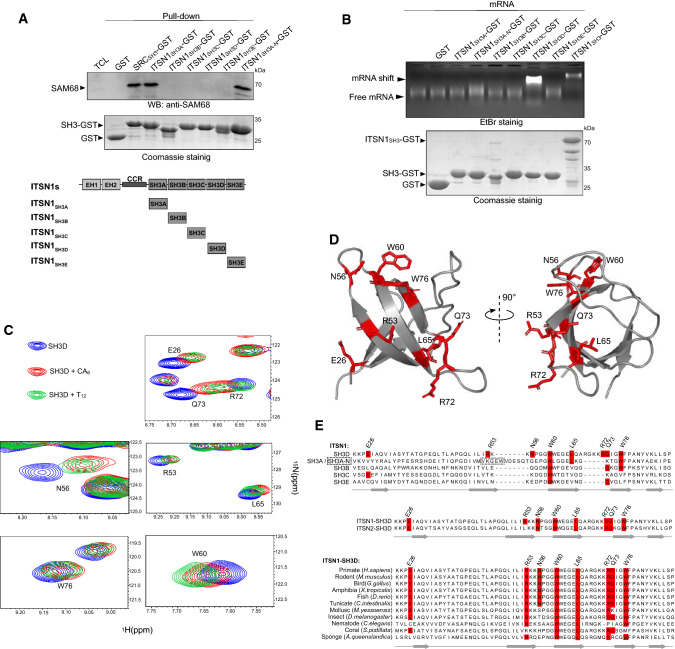
ITSN1 interacts with SAM68 and RNA via two different SH3 domains. **a** GST-pull-down assay revealing the interaction between ITSN1 SH3A domain and SAM68 in vitro. GST was used as a negative control, whereas SH3 domain of SRC kinase was used as a positive control. Immobilized GST-fused SH3 domains were used to precipitate SAM68 from HEK cell lysates. Proteins were visualized using Coomassie staining and Western blotting with anti-SAM68 antibody. **b** RNA mobility shift assay demonstrating the direct interaction between RNA and ITSN1 SH3D domain. Purified GST-fused ITSN1 SH3 domains or ITSN1_SH3_ were incubated with 0.4 pmol of 2Luc mRNA and resolved in agarose gel (upper panel). The same amounts of protein samples were resolved via SDS-PAGE and visualized using Coomassie (lower panel). **c** Two-dimensional ^1^H-^15^N SOFAST-HMQC spectra of ITSN1 SH3D domain in the presence of indicated ssDNA oligonucleotides (CA_6_ or T_12_). Eight residues with the major chemical shift perturbations were selected and are shown. **d** Structure of ITSN1 SH3D domain. Amino acid residues indicated on the ^1^H-^15^N SOFAST-HMQC spectra are shown and labeled. PDB structure 6GBU was used for the visualization. **e** Protein alignments showing the conservation of indicated amino acid residues in five ITSN1 SH3 domains (upper panel), ITSN1 and ITSN2 SH3D domains (middle panel), and SH3D domains of ITSN1 homologues in animals (lower panel)

While ITSN1 interacts with SAM68 through the SH3 domains (Figs. 1 and 2), these domains may also be involved in the binding of ITSN1 to mRNA (Fig. 3d, e and [56]). We, therefore, dissected whether SH3 domains were specifically involved in the binding to nucleic acids and SAM68 PRMs. The results of pull-down assays using purified SH3 domains indicate that SH3A and SH3A-N (the neuronal isoform of SH3A) are critical for the binding of ITSN1 to SAM68 (Fig. 4a), consistent with the leading role of SH3A in orchestrating the interaction with protein harboring PRMs [57]. The neuronal isoform of SH3A has an insertion of several amino acids that affect the specificity of the interaction between SH3A domain and its ligands in neurons [44]. As to whether a specific SH3 domain interacts with mRNA, only the binding of SH3D to mRNA was found to be significant (Fig. 4b). These results prompted us to identify whether specific residues in SH3D could explain this affinity for RNA. We, therefore, decided to analyze the interaction of SH3D with single stranded nucleic acids by NMR spectroscopy. After the resonance assignment of SH3D (see materials and methods), ^15^N-labeled SH3D domain were acquired in the presence or absence of short single stranded nucleic acids (12 T or 6 CA repeats, Fig. 4c, d, Supplementary Figure S8). Eight residues displayed major chemical shift differences in the presence of nucleic acids thus delineating the nucleic acid-interacting surface. Notably, residues L65, R72 and Q73 that are located on the two sides of an unstructured loop, are missing in the other SH3 domains of ITSN1 (Fig. 4e), which emphasizes the specific capacity of SH3D to bind to single stranded nucleic acids. In addition, most of these residues are conserved in the paralagous protein ITSN2 and largely across animal species (Fig. 4e).

The role of each SAM68 PRM in the interaction with ITSN1 was also examined. Using the microtubule bench with truncated forms of SAM68 as preys and ITSN1 SH3 domains as baits, we found that an N-terminal PRM of SAM68, P0 is leading the interaction with the SH3 domains of ITSN1 (Supplementary Figure S9).

### ITSN1 overexpression orchestrates SAM68 Nuclear Bodies dynamics in HeLa cells

**Fig. 5:**
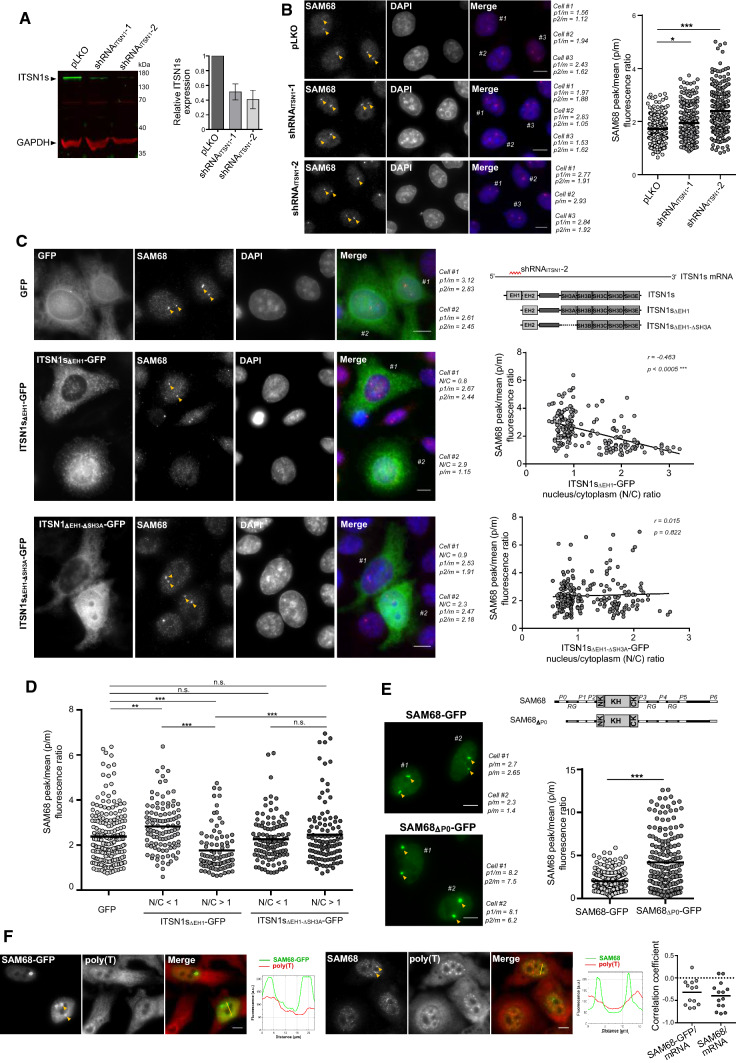
Dissociation of SAM68 nuclear bodies lacking mature mRNA is associated with expression of ITSN1 and nuclear accumulation of ectopic ITSN1s. **a** Western blot analysis representing the efficiency of ITSN1 knockdown in HeLa cells using shRNA. HeLa cells were transduced with lentiviral particles encoding two ITSN1-specific shRNAs (shRNA_ITSN1_-1 and shRNA_ITSN1_-2). Control cells were transduced with a nonsilencing virus (pLKO). Transduced cells were selected using puromycin (1 µl/ml) and were harvested for Western blot analysis 72 h after selection. The histogram represents the relative ITSN1s expression normalized to GAPDH expression from three experiments. **b** Immunofluorescent analysis of SAM68 nuclear bodies (SNBs) in HeLa cells under the condition of ITSN1 knockdown. Following viral transduction (pLKO, shRNA_ITSN1_-1, shRNA_ITSN1_-2) and antibiotic selection (puromycin, 1 µl/ml), cells were fixed and stained with anti-SAM68 antibodies. Randomly selected cells were analyzed for the mean SAM68 and maximum SNB fluorescent intensity as described in Materials and Methods and Supplementary Figure S10A. Peak/mean (p/m) fluorescence ratios for the labeled cells are indicated. The scatter plot (right panel) represents p/m fluorescence ratio values (*n* = 250 in each group) obtained in control and experimental HeLa cells (150–170 cells in each group from three experiments). SNBs are pointed with yellow arrows here and below. Scale bar: 15 µm. *p < 0.05, ***p < 0.0005, two-tailed *t* test. **c** Immunofluorescent analysis of SNBs in HeLa cells following the overexpression of shRNA_ITSN1_-2-resistant ITSN1 mutants. ITSN1 silencing was performed in HeLa cells as described above and in Materials and Methods. Two days after puromycin selection, cells were transiently transfected with GFP-, ITSN1s_ΔEH1_-GFP- or ITSN1s_ΔEH1ΔSH3A_-GFP-encoding plasmids, then fixed 24 h post-transfection and stained with anti-SAM68 antibodies. As shRNA_ITSN1_-2 antisense sequence is specific to the EH1-domain-encoding sequence of the ITSN1 mRNA, the mutants lacking the EH1 domain were used (top right panel). Cells were analyzed for the mean SAM68 and maximum SNB fluorescent intensity, as well as the nucleus/cytoplasm ITSN1-GFP fluorescence ratio, as described in Materials and Methods section and Supplementary Fig. 10. Scale bar: 15 µm. Peak/mean (p/m) fluorescence ratios for the labeled cells and nucleus/cytoplasm (N/C) ITSN1-GFP ratios are indicated. The correlation between p/m and N/C values for 140 cells in each experimental group of HeLa cells (200–220 p/m values per group) are visualized using corresponding scatter plots (right panels). Spearman’s correlation coefficients and *p* values are indicated. **d** Scatter plot representing SAM68 peak/mean fluorescence ratios in HeLa cells following ITSN1 knockdown with the subsequent expression of GFP or GFP-fused ITSN1 truncated forms – ITSN1s_ΔEH1_ and ITSN1s_ΔEH1ΔSH3A_. Each data point represents SAM68 p/m fluorescence ratio. The values are grouped according to the ITSN1 truncated forms, as well as their level of the accumulation in the nucleus (N/C values). **p < 0.005, ***p < 0.0005, *n.s.* not significant, two-tailed *t* test. **e** The analysis of the fluorescence intensities of SNBs formed by overexpressed wild-type and P0-lacking SAM68 fused to GFP. HeLa cells transfected with the corresponding constructs (SAM68-GFP and SAM68_ΔP0_-GFP) were analyzed via fluorescent microscopy as described in Materials and Methods and Supplementary Figure S10. Scale bar: 15 µm. The scatter plot (right panel) represents peak/mean (p/m) fluorescence ratio values (*n* = 185 in each group) obtained in transfected HeLa cells (~ 120 cells in each group from three experiments). ***p < 0.0005, two-tailed *t* test. **f** *In situ* hybridization analysis demonstrating the absence of mRNA in SAM68 nuclear bodies (SNBs) in HeLa cells. Endogenous SAM68 was detected using anti-SAM68 antibodies (left panel), whereas ectopic expression of SAM68 was obtained by transfecting HeLa cells with the SAM68-GFP plasmid (right panel). Fluorescent Cy2-conjugated poly(T) probes were used to detect mRNA. The line profile representing the fluorescence intensity from two channels is shown next to the respective microphotograph. Scale bar: 15 µm. Scatter plot (right panel) represents the co-localization level between SNBs and mRNA. Each data point represents a correlation coefficient between fluorescence intensities from red and green channels along the line crossing SNBs

Let us remind that SAM68 displays both a homogenous distribution in the nucleoplasm (Supplementary Figure S3 and [53, 54]) and accumulates in specific NBs in some cell lines ([40] and Supplementary Figure S3). We thus explored the notion that ITSN1 may regulate the ratio between the pools of SAM68 located in SAM68-rich NBs and in the nucleoplasm. To test this hypothesis, we measured the enrichment of SAM68 in NBs after knocking down the expression of ITSN1 with two different shRNAs in HeLa cells (Fig. 5a, b, Supplementary Figure S10A). Reducing ITSN1 expression level increases the presence of SAM68 in SNBs, suggesting that ITSN1 is truly a negative regulator of SAM68 NBs in the nucleus. To probe the putative role SH3A that controls the interaction between SAM68 and ITSN1, SAM68 enrichment in NBs was quantified upon knockdown of the endogenous ITSN1 protein and add-back of shRNA-2-resistant ITSN1 with or without SH3A (ITSN1s_ΔEH1_-GFP or ITSN1s_ΔEH1-ΔSH3A_-GFP, respectively). The analysis was performed at the single cell level to quantify the positive or negative correlation between ITSN1 expression level and SAM68 enrichment in NBs. Adding-back ITSN1 containing all its SH3 domains (ITSN1s_ΔEH1_-GFP) decreased the enrichment of SAM68 in NBs but not when SH3A (ITSN1s_ΔEH1-ΔSH3A_-GFP) has been truncated (Fig. 5c, d). When added-back ITSN1s_ΔEH1_-GFP was mainly cytoplasmic, an enrichment of SAM68 in NBs was detected (Fig. 5c, left panel, cell #1). In contrast, significant SAM68 depletion in NBs was observed in cells displaying a nuclear location of ITSN1s_ΔEH1_-GFP (Fig. 5c, left panel, cell #2). However, when SH3A was truncated, added-back ITSN1s_ΔEH1-ΔSH3A_-GFP, whatever nuclear or cytoplasmic, no longer interferes with SAM68 distribution (Fig. 5c, left panel, cell #2). Thereby, nuclear localization of ITSN1 harboring SH3A domain correlated with depletion of SAM68 in NBs (Fig. 5c right panel, 5D, Supplementary Figure S10B) Reciprocally, truncating P0, the PRM of SAM68 that interacts with SH3A, increases the presence of SAM68-GFP in NBs (Fig. 5e).

Finally, given that mRNA and ITSN1 act in synergy to increase SAM68 solubility in vitro (Fig. 3c), we wondered whether mRNA was present in SAM68 NBs. HeLa cells stained with poly(T) fluorescent probes revealed the absence of an accumulation of poly(A) mRNA in SAM68 NBs, whatever SAM68-GFP was expressed or not (Fig. 5f). This result confirms a previous observation in motor neurons, where co-staining for SAM68 and poly(A) mRNA did not reveal an mRNA enrichment in nuclear SAM68-rich domains [58]. Similarly, an accumulation of nuclear ITSN1, whatever endogenously expressed or expressed with a GFP label, was not detected in SAM68 NBs (Supplementary Figure S10C).

## Discussion

Keeping soluble RBPs that harbor self-adhesive property is a major challenge  for cells to sustain RNA biogenesis and mRNA processing in the nucleus and to prevent a dramatic formation of RBP condensates. We propose here, a mechanism based on the specific chaperoning of RBPs by structured domains present in nuclear proteins. Several nuclear proteins harbor multiple SH3 domains (listed in Supplementary Table 1). These domains can be considered as chaperone candidates owing to their characteristic beta-barrel structure, elevated solubility and affinity for PRMs, especially the RxxPxxP and PxxPxR sequences that are present in many RBPs (listed in Supplementary Table 2). The cellular and in vitro results obtained in this study point towards a specific interaction between ITSN1, a protein with five SH3 domains and SAM68 in cells (Fig. 1c). SAM68 harbors numerous PRMs allocated along the whole sequence and it is likely that both the number and distribution of PRM repeats are important determinants in the interaction between RBPs and SH3 domain-containing proteins [26, 27]. In addition to the multiplicity of PRMs, SAM68 possesses specific domains that could participate in the formation of condensates: an RNA-binding domain with an ability for multimerization (KH domain) [39, 59, 60], and LCDs flanking the KH domain (RG repeats) which are known to participate in multivalent interactions. Consistently, SAM68 is the main constituent of nuclear bodies (SNBs) which have accordingly a high proportion of proteins with intrinsically disorder regions [61]. Finally, the fact that SAM68 appeared both in nuclear SNBs and diffuse in the nucleoplasm is particularly suitable to detect the increase in SAM68 solubility that should lead to the partial dissociation of SNBs.

Together, the results obtained both in vitro and in cellular context are consistent with an interaction between ITSN1 SH3 domains and PRMs of SAM68 that keeps the fraction of SAM68 involved in this interaction in a soluble state. Interestingly, PRMs are in close proximity with RGG-rich sequences. Interactions between ITSN1 SH3 domains and SAM68 PRMs may neutralize the multimerization propensity of SAM68 by blocking the RGG domains. Besides the multiplicity of SH3 domains enabling weak interactions with SAM68 PRMs, the interaction between ITSN1 SH3A domain and P0, the N-terminal PRM of SAM68 appears to be essential (Fig. 4a and Supplementary Figure S9C and D). Although other isolated ITSN1 SH3 domains do not bind SAM68 (Fig. 4a), SH3A could initiate multiple interactions between these ITSN1 SH3 domains and other SAM68 PRMs.

**Fig. 6:**
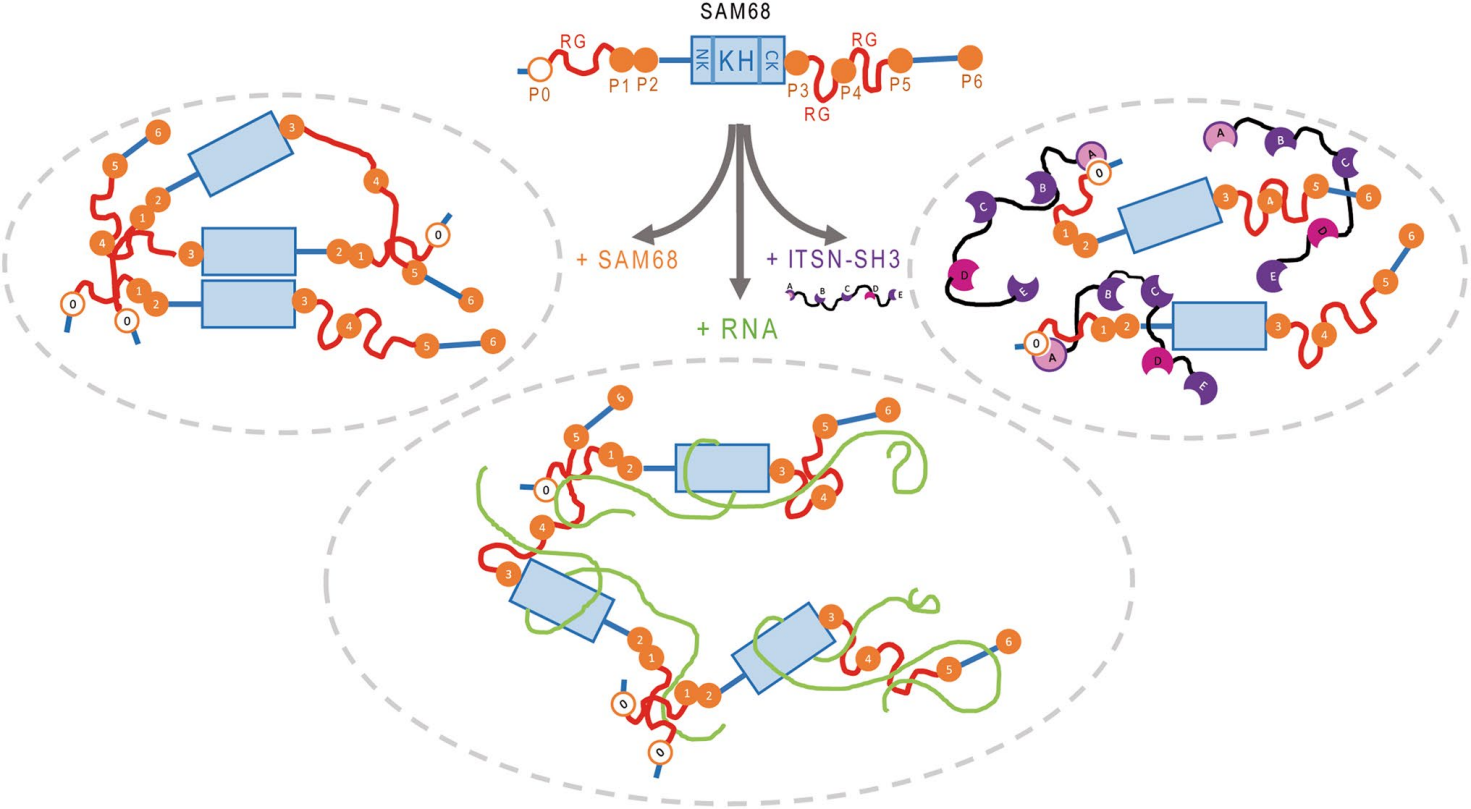
Schematic representation of the interplay between scaffold protein ITSN1 and RNA-binding protein SAM68. Due to the presence of RG repeats and KH domain that promote protein oligomerization and phase separation, SAM68 tends to form insoluble aggregates in vitro and SNBs in cells. Increasing the amount of mRNA favors the solubilization of SAM68, since RG repeats and KH domain are involved in mRNA binding. Stimulated by the interaction between SH3D and mRNA, ITSN1 promotes the solubilization of SAM68 through the interaction between ITSN1 SH3A and SAM68 P0

Another interesting finding lies in the direct interaction between mRNA and the SH3D domains of ITNS1, consistent with a putative affinity of SH3 domains for RNA [56] and leading to the reduction in the ITSN1 concentration required to solubilize SAM68 in the presence of mRNA (Fig. 3c). The eight residues in SH3D displaying the most significant chemical shift variations are not conserved in the other ITSN1 SH3 domains but are largely conserved across species pointing toward a specific function for this domain. In this context, the binding of SAM68 and ITSN1 with mRNA locally increases the occurrence of ITSN1 SH3A-SAM68 P0 complexation. The non-mutually exclusive interactions of ITSN1 SH3A and D domains with SAM68 and RNA revealed in this study thus supports the notion by which mRNA acts as a scaffold to initiate interaction between RBPs and their chaperones (Fig. 6). In support of this notion, no poly(A) RNA enrichment was detected in SAM68 NBs in HeLa cells by in situ hybridization (Fig. 5f). The absence of poly(A) RNA enrichment in SNBs, also observed in wild-type motor neurons [58], may, therefore, be consecutive to a phase separation between (i) a SAM68-rich and RNA-poor phase, i.e., the SNBs and (ii) an mRNA and ITSN1-rich phase in which SAM68 is soluble, i.e., the nucleoplasm.

Accordingly, to date, no metabolic activity has been detected in SNBs and some partners essential for the activity of SAM68 like U1snRNP are not identified as constituting elements of SNBs [62]. SNBs could be passive storage site of SAM68 when SAM68 concentration is above the solubilization, and can provide a fine control of SAM68 concentration in the nucleoplasm. Post-translational modifications is also a possible mean to regulate the pool of soluble SAM68 in a similar way that the post-translational modifications in FUS RGG regulate its capacity to form condensates [63]. In agreement with this, phosphorylation, methylation, acetylation and sumoylation have been reported for SAM68 (reviewed in [64]).

How ITSN1 solubilizes a measurable fraction of SAM68, owing to its low level of expression compared to SAM68 in cells [65] and in most human tissues [66], remains, however, surprising. A stable bipartite complex (ITSN1/SAM68) should only solubilize a tiny fraction of SAM68 (less than 1/100). Several scenarios are nevertheless possible: (i) ITSN1 interacts transiently with SAM68 (which is very likely) to allow the proper folding of SAM68 or to favor the recruitment of other factors required for the SAM68 solubility such as mRNA, (ii) A transient interaction between ITSN1 and SAM68 would allow the phosphorylation of SAM68 which would afterward maintain SAM68 solubility in the nucleoplasm.

Interestingly, mRNA recognition by the SH3D of ITSN1 together with SAM68, may help to recruit specific mRNAs to regulate their splicing. This could notably be critical in multiple cancer types, where SAM68 is overexpressed and regulates expression and alternative splicing of several proto-oncogenes [64] but also in neurons that require a complex splicing regulation to exert their higher functions and in which ITSN1 is abundantly expressed, while its disruption causes deficiency of learning and memory [67]. On its side, SAM68 has been also associated with neurogenesis and spinal muscular atrophy (SMA) [68, 69], possibly via the splicing of pre-mRNA transcripts critically involved in human neuronal diseases such as *SMN2*. In addition, the presence of large intranuclear SAM68 aggregates in brain sections of patients affected by Fragile X-associated Tremor/Ataxia Syndrome (FXTAS) has been confirmed and linked also with altered splicing functions [70]. A genome wide analysis focused on the possible role of ITSN1 in mRNA splicing in neurons should clarify whether ITSN1 is a splicing regulator and whether a functional link with SAM68 functions can be identified. We anticipate that the mechanistic model developed in this study will provide basis for further investigations on the consequences of PRMs/SH3 domains interactions on the processing of specific mRNA by RBPs such as SAM68 involved in human diseases.

## Electronic supplementary material

Below is the link to the electronic supplementary material.

Supplementary material 1 (PDF 11189 kb)
