# Supplementary material for: ITSN1 regulates SAM68 solubility through SH3 domain interactions with SAM68 proline-rich motifs
Source: Cell Mol Life Sci. 2020 Aug 11;78(4):1745–63. doi: 10.1007/s00018-020-03610-y (PMC7904728; doi:10.1007/s00018-020-03610-y)
Supplement: Supplementary file 1 — Supplementary material 1 (PDF 11189 kb) [file 18_2020_3610_MOESM1_ESM.pdf]

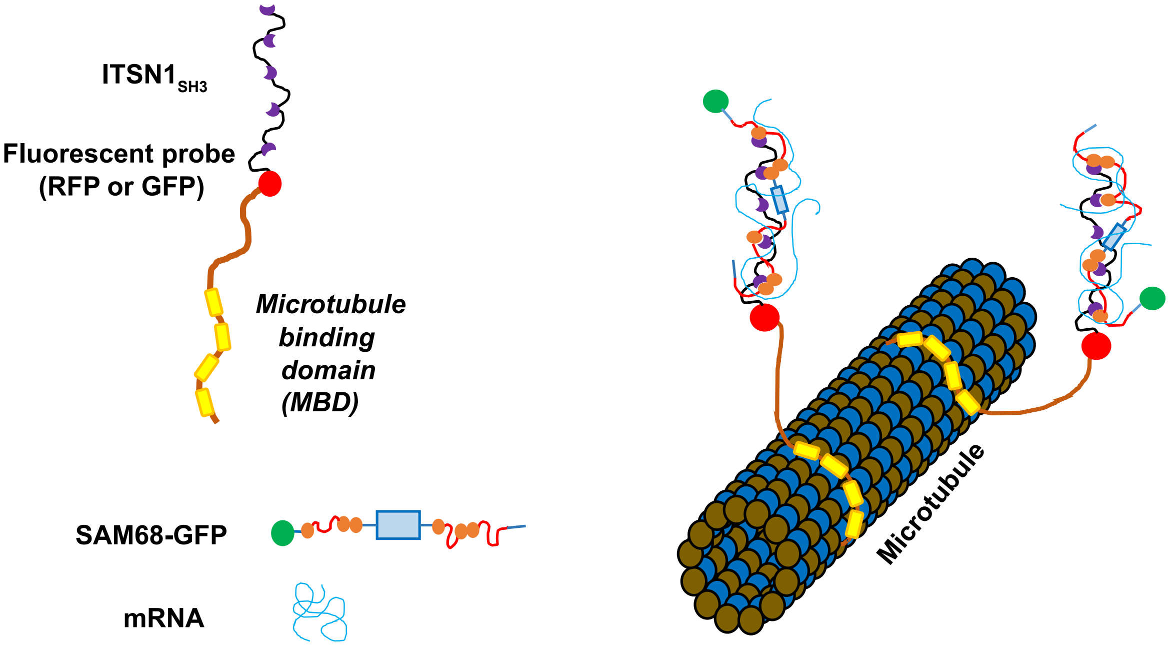

Microtubule bench principle

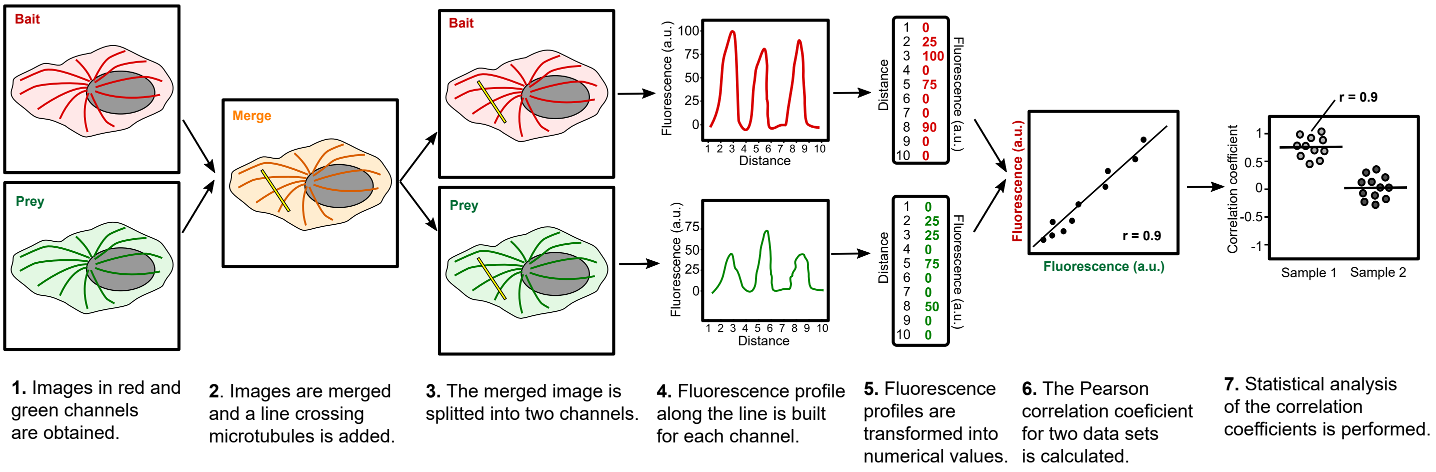

Calculation of the correlation coefficient

Supplementary Figure S1

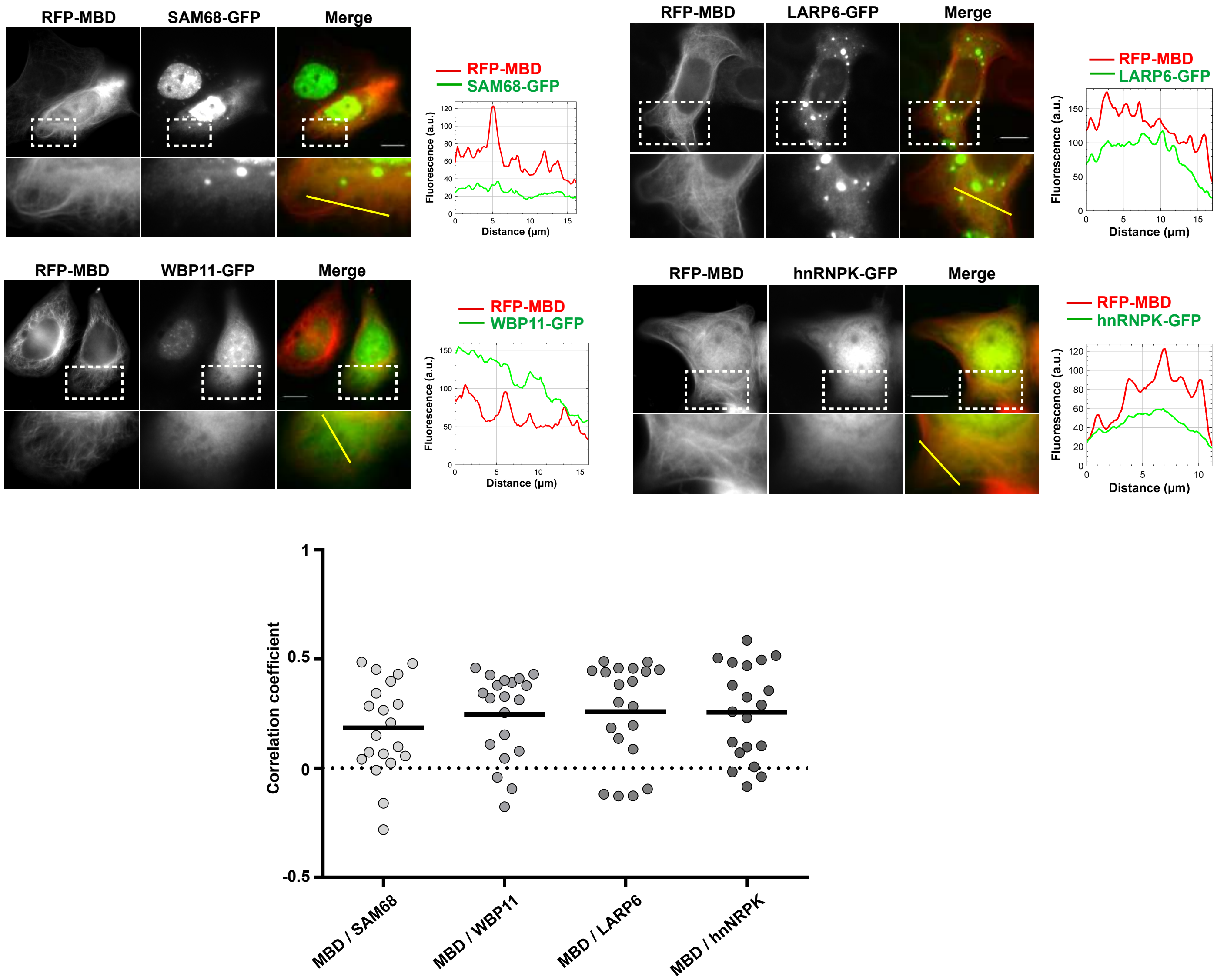

Supplementary Figure S2

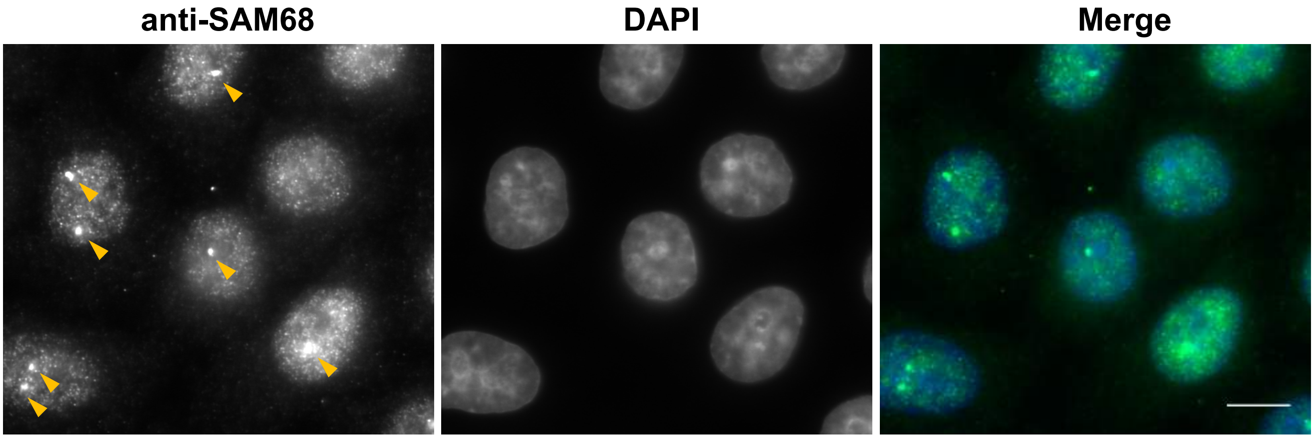

Supplementary Figure S3

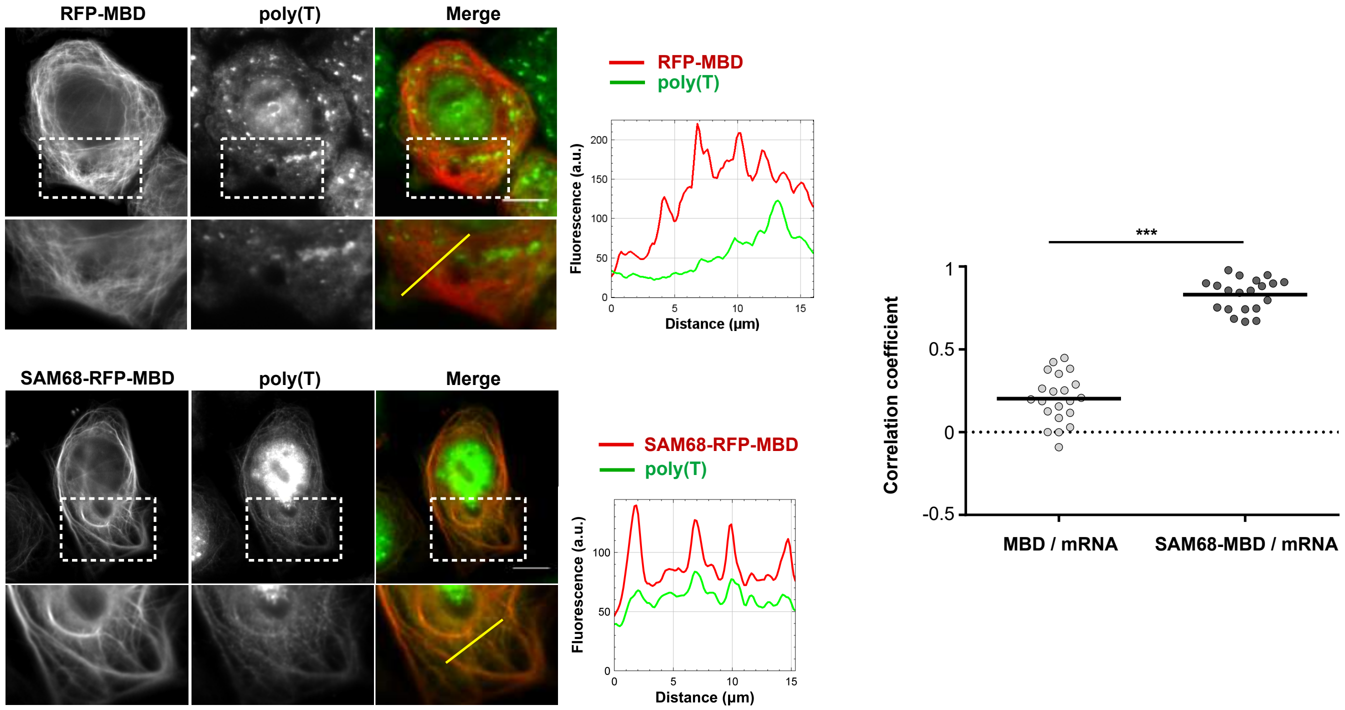

Supplementary Figure S4

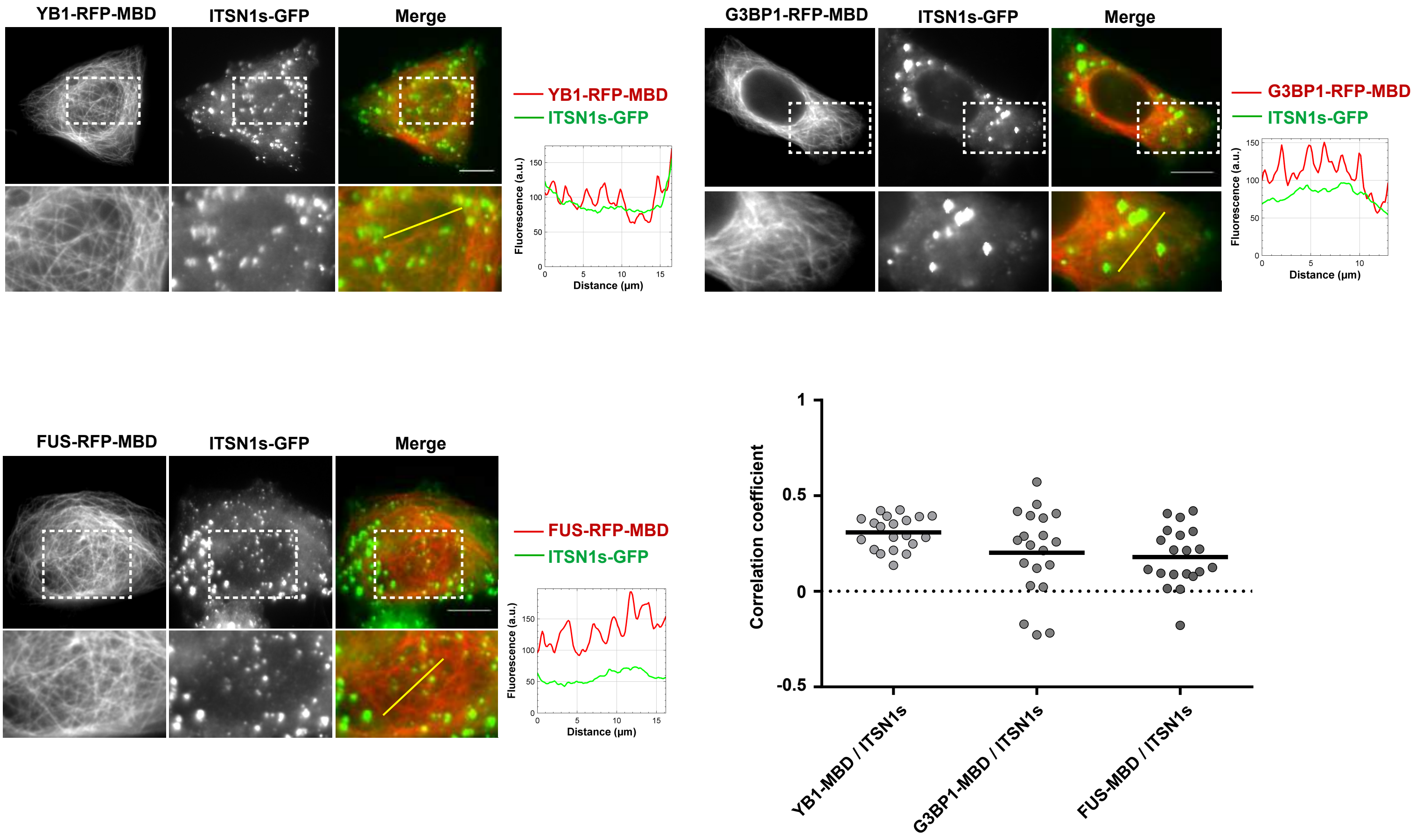

Supplementary Figure S5

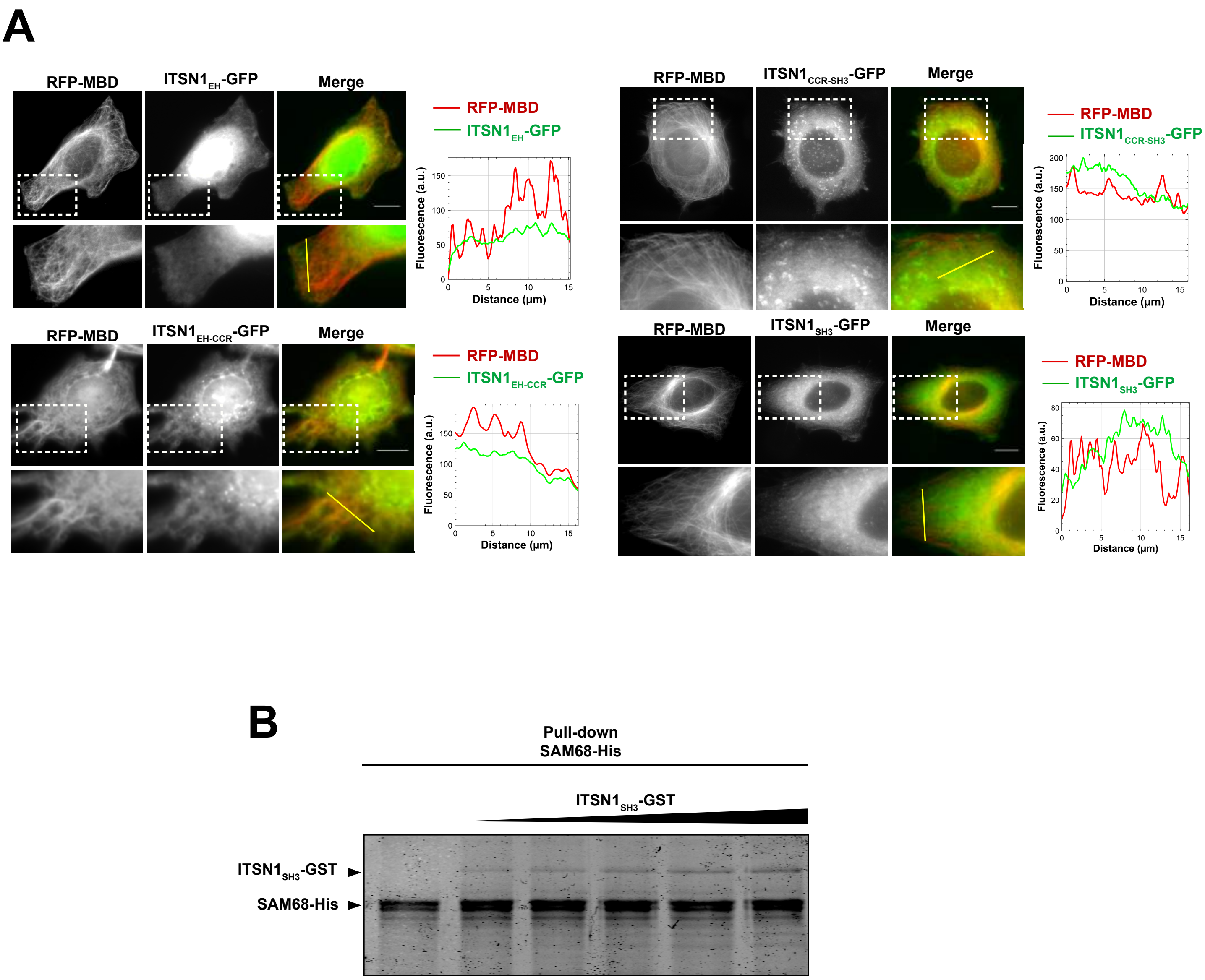

Supplementary Figure S6

A

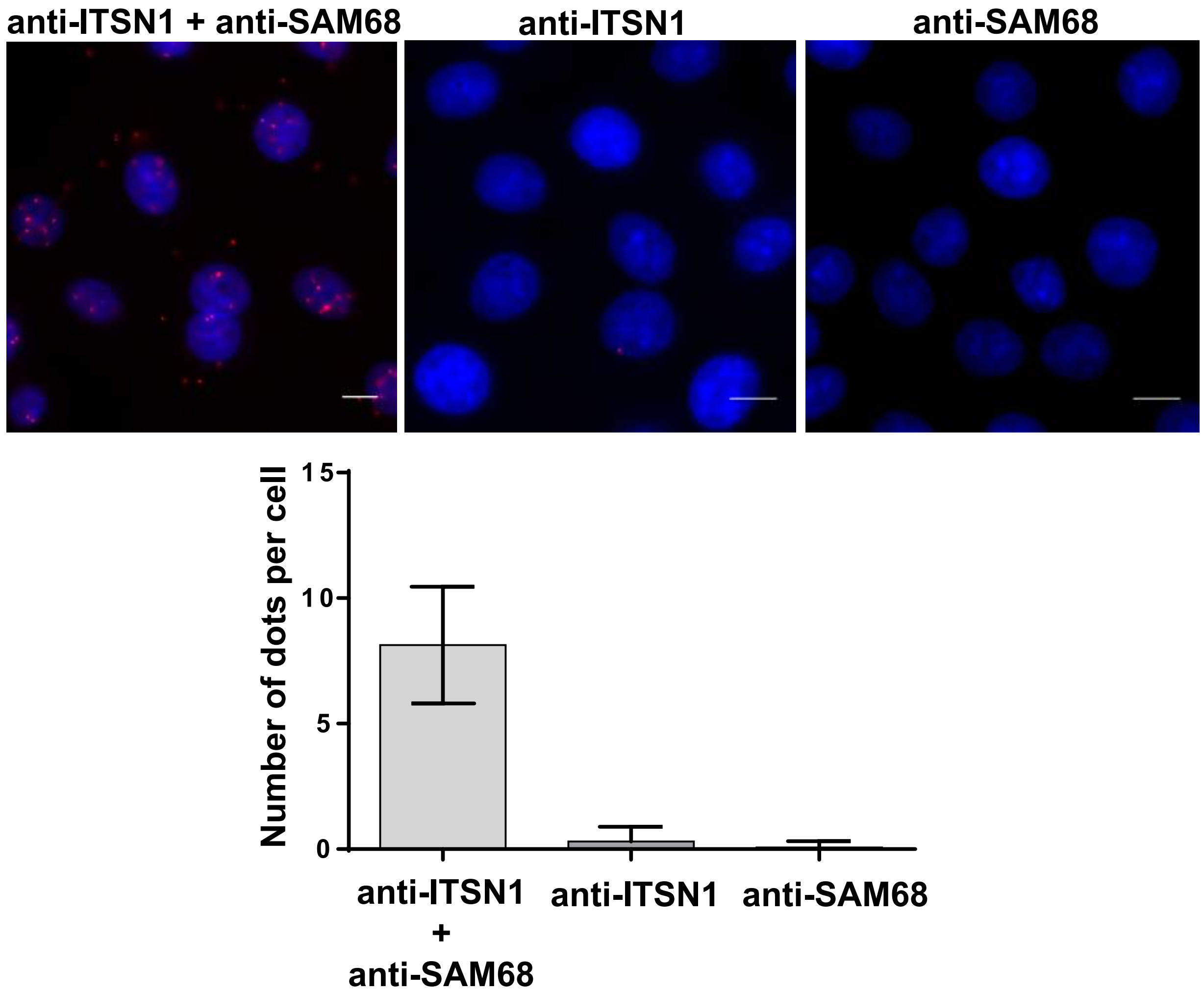

B

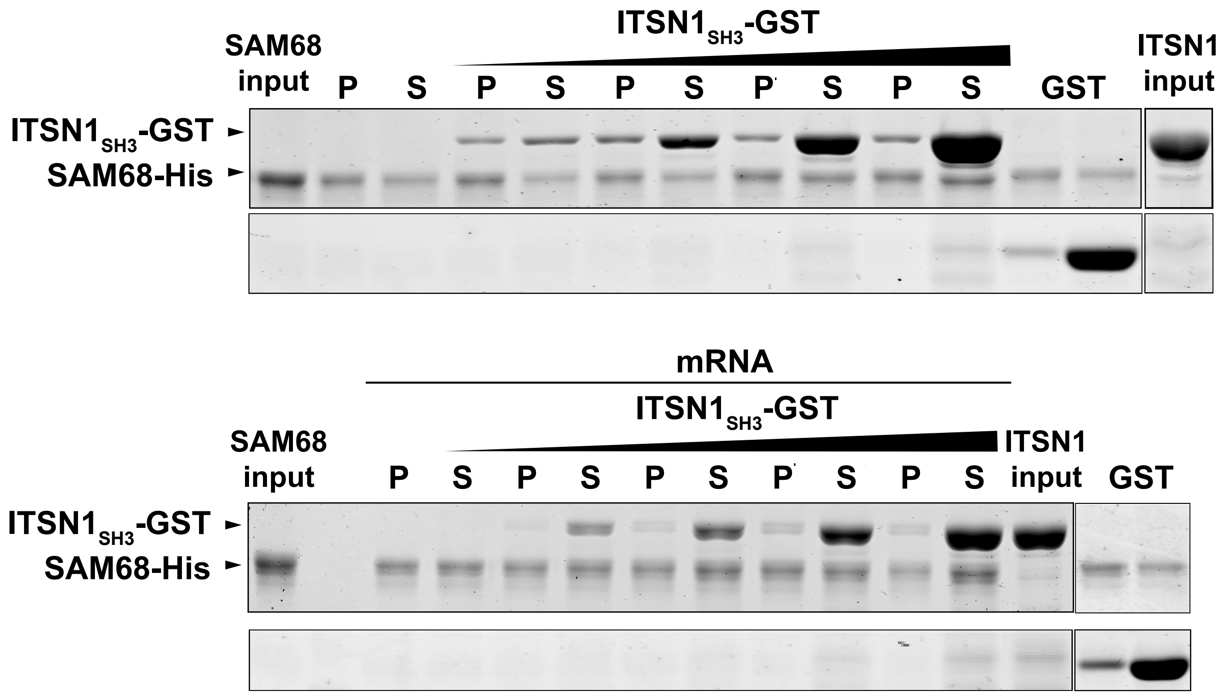

Supplementary Figure S7

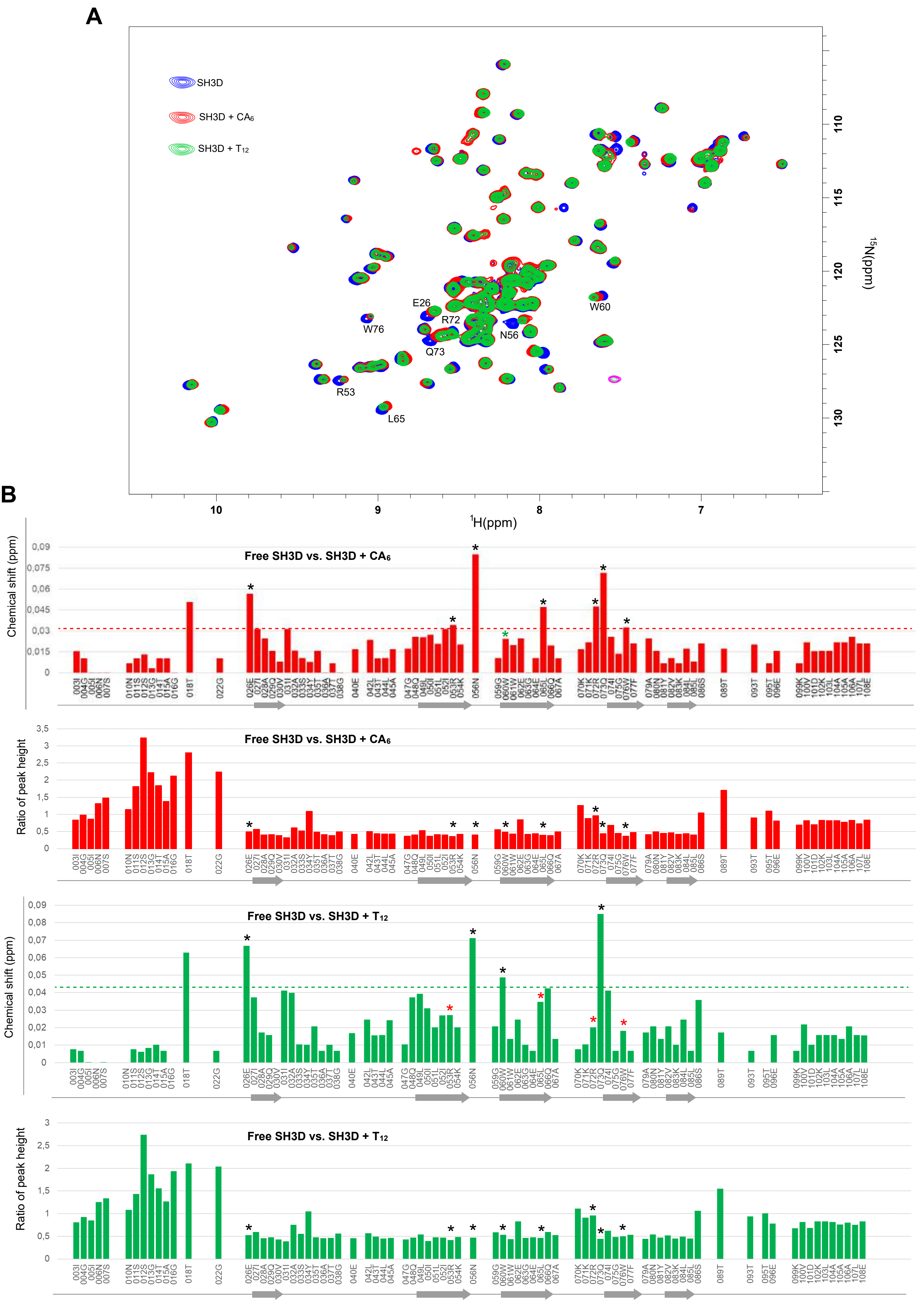

Supplementary Figure S8

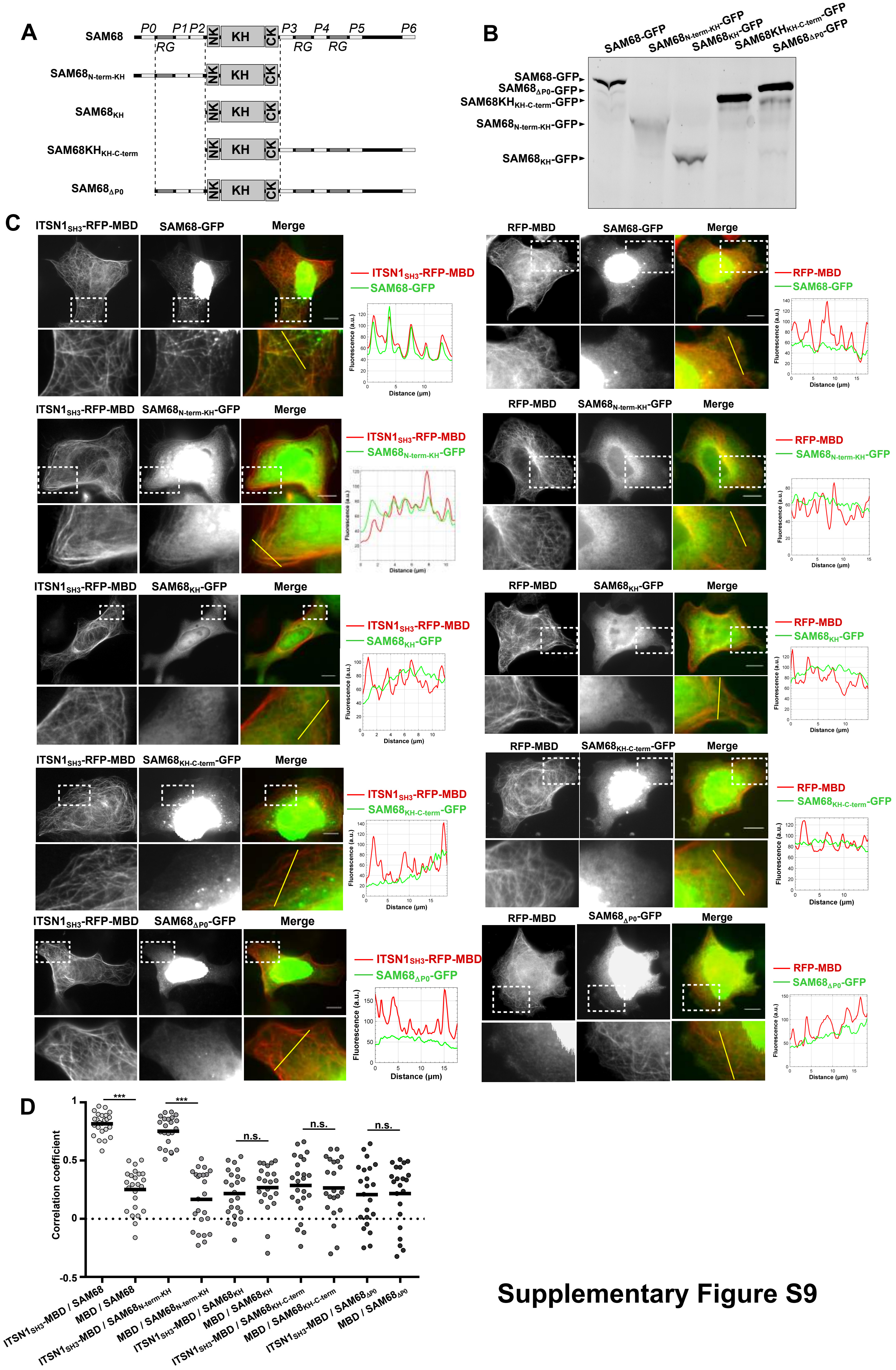

Supplementary Figure S9

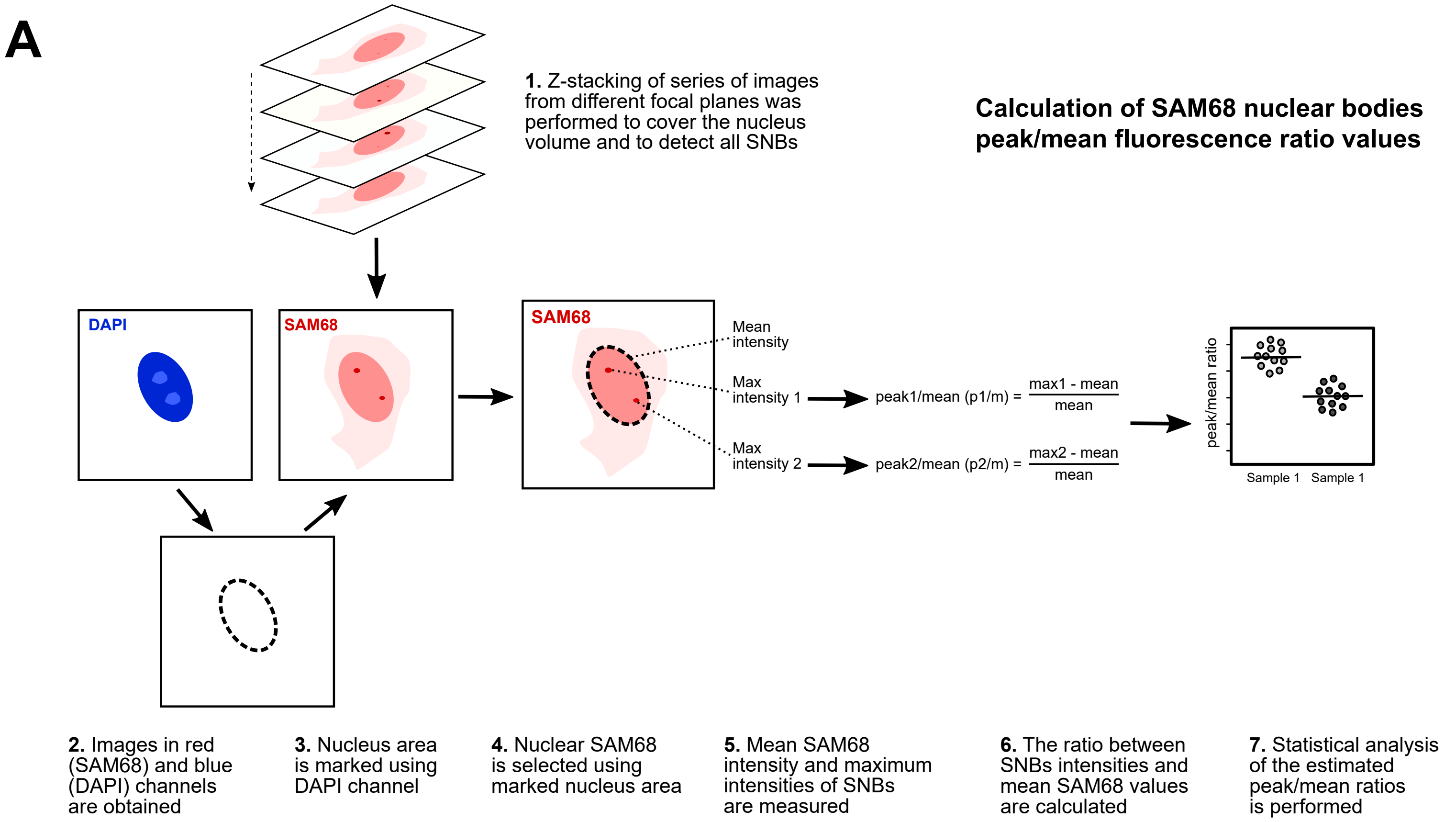

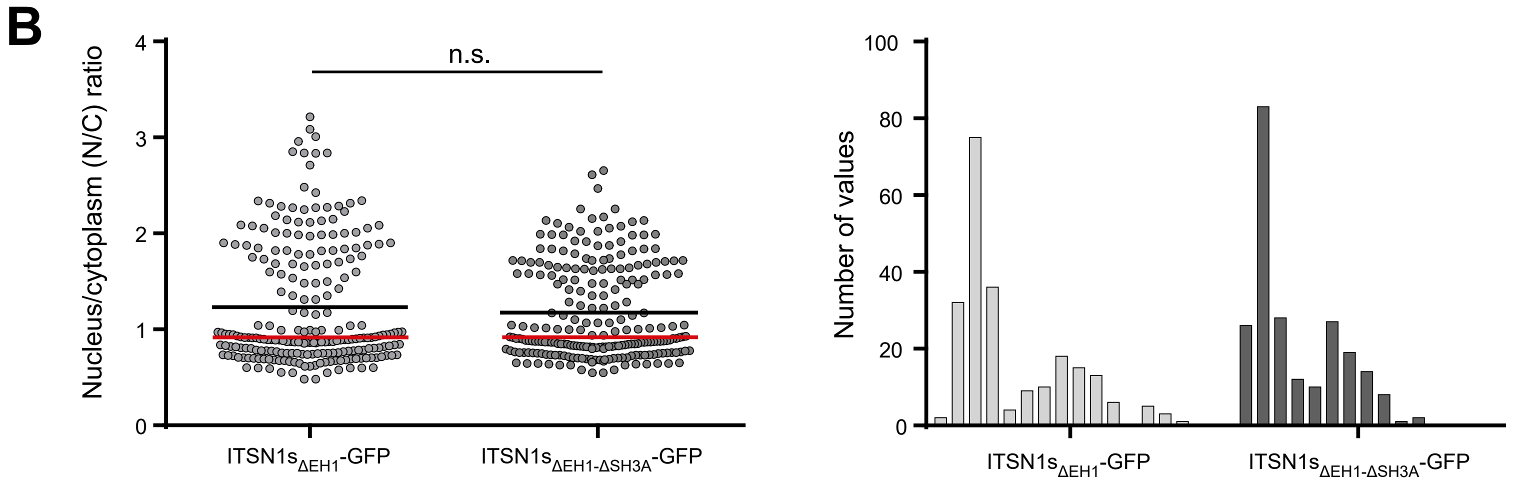

The scatter plot of variation, mean (black line), and median (red line) values of nucleus/cytoplasm (N/C) ratios for ITSN1s-GFP constructs. n.s. - non-significant.

The frequency distribution histogram of nucleus/cytoplasm (N/C) ratios for ITSN1s-GFP constructs.

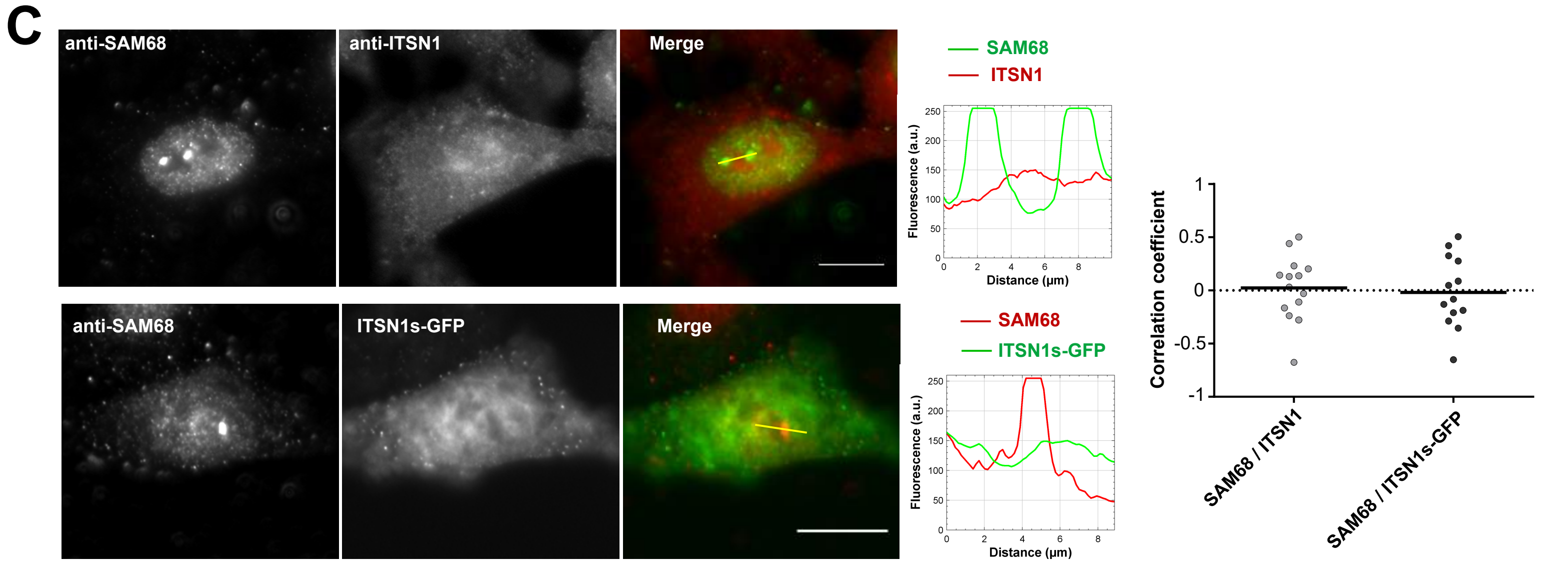

Supplementary Figure S10

Supplementary Table 1. The list of human proteins containing three or more SH3 domains							
**NCBI gene ID**	**Gene symbol**	**Full name**	**Other names**	**NCBI protein ID**	**Number of SH3 domains**	**Nuclear localization**	**Nuclear localization evidence**
23268	DNMBP	dynamin binding protein	TUBA; CTRCT48; ARHGEF36	NP_056036.1	6	Found	Uhlen et al., 2010
6453	ITSN1	intersectin 1	ITSN; SH3D1A; SH3P17	NP_003015.2	5	Found	Alvici et al., 2018; Uhlen et al., 2010
50618	ITSN2	intersectin 2	SWA; SWAP; SH3D1B; SH3P18; PRO2015	NP_006268.2	5	Not found	
152503	SH3D19	SH3 domain containing 19	EBP; EVE1; Kryn; Eve-1; SH3P19	NP_001009555.3	5	Found	Uhlen et al., 2010
9644	SH3PXD2A	SH3 and PX domains 2A	FISH; TKS5; SH3MD1	NP_055446.2	5	Not found	
285590	SH3PXD2B	SH3 and PX domains 2B	FTHS; HOFI; TKS4; TSK4; FAD49; KIAA1295	NP_001017995.1	4	Found	Uhlen et al., 2010
57630	SH3RF1	SH3 domain containing ring finger 1	POSH; RNF142; SH3MD2	NP_065921.2	4	Not found	
344558	SH3RF3	SH3 domain containing ring finger 3	POSH2; SH3MD4	NP_001092759.1	4	Found	Uhlen et al., 2010
23504	RIMBP2	RIMS binding protein 2	RBP2; RIM-BP2; PPP1R133	NP_001338155.1	3	Not found	
85376	RIMBP3	RIMS binding protein 3	RIM-BP3; RIMBP3A; RIMBP3.1; RIM-BP3.1; RIM-BP3.A	NP_056487.1	3	Not found	
9256	TSPOAP1	TSPO associated protein 1	PRAX1; BZRAP1; PBR-IP; PRAX-1; RIMBP1; RIM-BP1	NP_004749.2	3	Not found	
23607	CD2AP	CD2 associated protein	CMS	NP_036252.1	3	Not found	
4690	NCK1	NCK adaptor protein 1	NCK; nck-1; NCKalpha	NP_006144.1	3	Found	Lawe, Hahn, & Wong, 1997
8440	NCK2	NCK adaptor protein 2	GRB4; NCKbeta	NP_003572.2	3	Found	Jahn et al., 2001
79729	SH3D21	SH3 domain containing 21	C1orf113	NP_001156002.1	3	Found	Uhlen et al., 2010
30011	SH3KBP1	SH3 domain containing kinase binding protein 1	HSB1; AGMX2; CIN85; GIG10; HSB-1; IMD61; MIG18; CD2BP3	NP_001340821.1	3	Not found	
153769	SH3RF2	SH3 domain containing ring finger 2	HEPP1; POSHER; RNF158; PPP1R39	NP_689763.4	3	Found	Kim et al., 2013; Uhlen et al., 2010
10580	SORBS1	sorbin and SH3 domain containing 1	CAP; FLAF2; R85FL; SH3D5; SORB1; SH3P12	NP_001030126.1	3	Found	Lebre et al., 2001; Uhlen et al., 2010
8470	SORBS2	sorbin and SH3 domain containing 2	ARGBP2; PRO0618	NP_001257700.1	3	Found	Wang, Golemis, & Kruh, 1997; Uhlen et al., 2010
10174	SORBS3	sorbin and SH3 domain containing 3	SCAM1; SH3D4; SCAM-1; vinexin	NP_005766.3	3	Found	Uhlen et al., 2010

Supplementary Table 2. The list of human RNA-binding proteins containing more than five proline motifs										
**NCBI gene ID**	**Gene symbol**	**Full name**	**Other names**	**NCBI protein ID**	**RNA-binding domain**	**Number of PxxP motifs**	**Number of RxxPxxP motifs**	**Number of PxxPxR motifs**	**Database**	**Nuclear localization**
4940	OAS3	2'-5'-oligoadenylate synthetase 3	p100; p100OAS	NP_006178.2	2-5-oligoadenylate_synth	7	0	0	QuickGO	Found
22803	XRN2	5'-3' exoribonuclease 2		NP_001304889.1	5_3_exoribonuclease	5	1	0	QuickGO	Found
54464	XRN1	5'-3' exoribonuclease 1	SEP1	NP_061874.3	5_3_exoribonuclease	7	0	0	QuickGO	Not found
23536	ADAT1	adenosine deaminase tRNA specific 1	HADAT1	NP_036223.2	A_deamin	5	0	0	QuickGO	Found
2058	EPRS	glutamyl-prolyl-tRNA synthetase	EARS; PARS; QARS; QPRS; HLD15; PIG32; GLUPRORS	NP_004437.2	aa-tRNA-synth	9	0	0	QuickGO	Not found
54913	RPP25	ribonuclease P and MRP subunit p25		NP_060263.2	Alba	6	0	0	QuickGO	Found
9716	AQR	aquarius intron-binding spliceosomal factor	IBP160; fSAP164	NP_055506.1	Aquarius_N	5	0	0	QuickGO	Found
4076	CAPRIN1	cell cycle associated protein 1	M11S1; GPIAP1; RNG105; GPIP137; GRIP137; p137GPI	NP_005889.3	Caprin-1_C	6	1	1	QuickGO	Not found
22794	CASC3	CASC3 exon junction complex subunit	BTZ; MLN51	NP_031385.2	CASC3	12	1	2	QuickGO	Found
51087	YBX2	Y-box binding protein 2	BPC; MSY2; CSDA3; CONTRIN	NP_057066.2	CSD	8	2	1	RBPDB/AttRACT	Found
27254	CSDC2	cold shock domain containing C2	PIPPIN; dJ347H13.2	NP_055275.1	CSD	5	0	0	RBPDB	Found
7157	TP53	tumor protein p53	P53; BCC7; LFS1; BMFS5; TRP53	NP_000537.3	CTD	6	0	0	QuickGO	Found
104	ADARB1	adenosine deaminase RNA specific B1	RED1; ADAR2; DRABA2; DRADA2	NP_056648.1	dsRBD	5	0	0	QuickGO	Found
3609	ILF3	interleukin enhancer binding factor 3	CBTF; DRBF; MMP4; MPP4; NF90; NFAR; NF110; NF90a; NF90b; NF90c; NFAR2; TCP80;	NP_060090.2	dsRBD	5	0	0	QuickGO	Found

			DRBP76; NF110b; NFAR-1; NFAR-2; NFAR90; TCP110; MPP4110; NF90ctv; NFAR110; MPHOSPH4; NF-AT-90							
6780	STAU1	staufen double-stranded RNA binding protein 1	STAU; PPP1R150	NP_001306064.1	dsRBD	6	0	0	QuickGO	Not found
23405	DICER1	dicer 1, ribonuclease III	DCR1; GLOW; MNG1; Dicer; HERNA; RMSE2; Dicer1e; K12H4.8-LIKE	NP_085124.2	dsRBD; PAZ	9	0	1	QuickGO	Not found
54487	DGCR8	DGCR8 microprocessor complex subunit	Gy1; pasha; DGCRK6; C22orf12	NP_073557.3	dsRBDx2	7	0	0	QuickGO	Found
27067	STAU2	staufen double-stranded RNA binding protein 2	39K2; 39K3	NP_001157852.1	dsRBDx4	5	0	0	QuickGO	Found
6651	SON	SON DNA binding protein	SON3; BASS1; DBP-5; NREBP; TOKIMS; C21orf50	NP_620305.2	DSRM	36	0	0	QuickGO	Found
103	ADAR	adenosine deaminase RNA specific	DSH; AGS6; G1P1; IFI4; P136; ADAR1; DRADA; DSRAD; IFI-4; K88DSRBP	NP_001102.3	DSRM; ADEAMc	6	0	0	AttRACT/QuickGO	Found
1660	DHX9	DExH-box helicase 9	LKP; RHA; DDX9; NDH2; NDHII	NP_001348.2	DSRM; HrpA	5	0	0	AttRACT/QuickGO	Found
23293	SMG6	SMG6 nonsense mediated mRNA decay factor	EST1A; SMG-6; C17orf31; hSMG5/7a	NP_060045.4	EST1; PIN	8	0	0	QuickGO	Found
26065	LSM14A	LSM14A mRNA processing body assembly factor	RAP55; FAM61A; RAP55A; C19orf13	NP_001107565.1	FDF	6	0	2	QuickGO	Not found
92345	NAF1	nuclear assembly factor 1 ribonucleoprotein		NP_612395.2	Gar1/Naf1	10	1	0	QuickGO	Found
317781	DDX51	DEAD-box helicase 51		NP_778236.2	Helicase	9	0	0	QuickGO	Found
9704	DHX34	DExH-box helicase 34	HRH1; DDX34	NP_055496.2	Helicase	9	2	0	QuickGO	Found
11325	DDX42	DEAD-box helicase 42	RHELP; RNAHP; SF3B8; DDX42P; SF3b125	NP_031398.2	Helicase	8	0	1	QuickGO	Found

79039	DDX54	DEAD-box helicase 54	DP97	NP_001104792.1	Helicase	6	1	0	QuickGO	Found
64794	DDX31	DEAD-box helicase 31	PPP1R25	NP_073616.6	Helicase	5	1	0	QuickGO	Found
9785	DHX38	DEAH-box helicase 38	RP84; DDX38; PRP16; PRPF16	NP_054722.2	Helicase	5	1	1	QuickGO	Found
22907	DHX30	DExH-box helicase 30	DDX30; RETCOR; NEDMIAL	NP_619520.1	Helicase	5	1	1	QuickGO	Not found
6832	SUPV3L1	Suv3 like RNA helicase	SUV3	NP_003162.2	Helicase; SUV3	5	0	1	AttRACT/QuickGO	Found
26057	ANKRD17	ankyrin repeat domain 17	GTAR; MASK2; NY-BR-16	NP_115593.3	KH	20	0	1	RBPDB/QuickGO	Found
22889	KHDC4	KH domain containing 4, pre-mRNA splicing factor	BLOM7; KIAA0907; SNORA80EHG	NP_055764.2	KH	12	0	0	QuickGO	Found
10657	KHDRBS1	KH RNA binding domain containing, signal transduction associated 1	p62; p68; Sam68	NP_006550.1	KH	8	1	1	RBPDB/AttRACT/QuickGO	Found
54882	ANKHD1	ankyrin repeat and KH domain containing 1	MASK; MASK1; VBARP; PP2500	NP_060217.1	KH	14	0	1	RBPDB/AttRACT/QuickGO	Not found
8165	AKAP1	A-kinase anchoring protein 1	AKAP; PRKA1; AKAP84; TDRD17; AKAP121; AKAP149; D-AKAP1; PPP1R43; SAKAP84	NP_003479.1	KH	8	0	0	RBPDB/AttRACT/QuickGO	Not found
202559	KHDRBS2	KH RNA binding domain containing, signal transduction associated 2	SLM1; SLM-1	NP_001337551.1	KH	5	2	2	RBPDB/AttRACT	Not found
7536	SF1	splicing factor 1	BBP; MBBP; ZFM1; ZNF162; D11S636; ZCCHC25	NP_004621.2	KH; Znf_CCHC	24	0	0	RBPDB/AttRACT/QuickGO	Found
9513	FXR2	FMR1 autosomal homolog 2	FXR2P; FMR1L2	NP_004851.2	KHx2	6	2	0	RBPDB/AttRACT/QuickGO	Not found
92312	MEX3A	mex-3 RNA binding family member A	RKHD4; MEX-3A; RNF162	NP_001087194.1	KHx2; Znf_CCCH	11	2	0	RBPDB/QuickGO	Not found
399664	MEX3D	mex-3 RNA binding family member D	MEX3; TINO; RKHD1; MEX-3D; RNF193; OK/SW-cl.4	NP_976049.3	KHx2; Znf_RING	15	2	1	RBPDB/QuickGO	Found
84206	MEX3B	mex-3 RNA binding family member B	RKHD3; MEX-3B; RNF195	NP_115622.2	KHx2; Znf_RING	6	0	0	RBPDB/QuickGO	Found
51320	MEX3C	mex-3 RNA binding family member C	RKHD2; BM-013; MEX-3C; RNF194	NP_057710.3	KHx2; Znf_RING	13	2	1	RBPDB/QuickGO	Not found

3190	HNRNPK	heterogeneous nuclear ribonucleoprotein K	AUKS; CSBP; TUNP; HNRPK	NP_002131.2	KHx3	5	3	3	RBPDB/AttRACT/QuickGO	Found
80114	BICC1	BicC family RNA binding protein 1	BICC; CYSRD	NP_001073981.1	KHx3; SAM	5	0	0	RBPDB/QuickGO	Found
8570	KHSRP	KH-type splicing regulatory protein	p75; FBP2; KSRP; FUBP2	NP_003676.2	KHx4	18	1	0	RBPDB/AttRACT/QuickGO	Found
8880	FUBP1	far upstream element binding protein 1	FBP; FUBP; hDH V	NP_001290362.1	KHx4	14	0	0	RBPDB/QuickGO	Found
23367	LARP1	La ribonucleoprotein domain family member 1	LARP	NP_056130.2	La	15	1	1	RBPDB/QuickGO	Found
55132	LARP1B	La ribonucleoprotein domain family member 1B	LARP2	NP_060548.2	La	7	0	1	RBPDB/QuickGO	Found
23185	LARP4B	La ribonucleoprotein domain family member 4B	LARP5; KIAA0217	NP_001338206.1	La; RRM	8	0	0	RBPDB/QuickGO	Not found
113251	LARP4	La ribonucleoprotein domain family member 4	PP13296	NP_055970.1	La; RRM	5	0	0	RBPDB/QuickGO	Not found
6628	SNRPB	small nuclear ribonucleoprotein polypeptides B and B1	COD; CCMS; SNRPB1; SmB/B'; Sm-B/B'; snRNP-B; SmB/SmB'	NP_937859.1	LSm	6	1	0	RBPDB/QuickGO	Found
6638	SNRPN	small nuclear ribonucleoprotein polypeptide N	SMN; PWCR; SM-D; sm-N; RT-LI; HCERN3; SNRNP-N; SNURF-SNRPN	NP_003088.1	LSm	6	1	0	RBPDB	Found
134353	LSM11	LSM11, U7 small nuclear RNA associated		NP_775762.1	LSm	5	1	0	RBPDB/QuickGO	Found
4686	NCBP1	nuclear cap binding protein subunit 1	NCBP; Sto1; CBP80	NP_001338433.1	MIF4G-like	5	0	0	QuickGO	Found
190	NR0B1	nuclear receptor subfamily 0 group B member 1	AHC; AHX; DSS; GTD; HHG; AHCH; DA; DAX-1; NROB1; SRXY	NP_000466.2	NR_Repeatx4	5	1	0	QuickGO	Found
65083	NOL6	nucleolar protein 6	NRAP; UTP22; bA311H10.1	NP_075068.2	Nrap	9	1	1	QuickGO	Found
219988	PATL1	PAT1 homolog 1, processing body mRNA decay factor	Pat1b; hPat1b	NP_689929.2	Pat-C	7	0	1	QuickGO	Found
55124	PIWIL2	piwi like RNA-mediated gene silencing 2	CT80; HILI; mili; PIWIL1L	NP_001129193.1	PAZ	6	0	0	QuickGO	Found
23381	SMG5	SMG5 nonsense mediated mRNA decay factor	EST1B; SMG-5; LPTSRP1; LPTS-RP1	NP_056142.2	PIN	6	0	0	QuickGO	Found

9698	PUM1	pumilio RNA binding family member 1	PUMH; HSPUM; PUMH1; PUML1; SCA47	NP_001018494.1	PUM	6	0	0	RBPDB/AttRACT/QuickGO	Found
10250	SRRM1	serine and arginine repetitive matrix 1	160-KD; POP101; SRM160	NP_001290377.1	PWI	16	8	4	RBPDB/QuickGO	Found
3508	IGHMBP2	immunoglobulin mu DNA binding protein 2	HCSA; HMN6; CATF1; CMT2S; SMARD1; SMUBP2; ZFAND7	NP_002171.2	R3H; Znf_AN1	5	0	0	AttRACT/QuickGO	Found
29102	DROSHA	drosha ribonuclease III	RN3; ETOHI2; RNASEN; RANSE3L; RNASE3L; HSA242976	NP_037367.3	RIBOc; Rnc	24	1	1	QuickGO	Found
60528	ELAC2	elaC ribonuclease Z 2	ELC2; HPC2; COXPD17	NP_060597.4	RibonucZ	5	0	0	QuickGO	Found
5442	POLRMT	RNA polymerase mitochondrial	APOLMT; MTRNAP; MTRPOL; h-mtRPOL	NP_005026.3	RNA_POL	10	2	0	QuickGO	Not found
5430	POLR2A	RNA polymerase II subunit A	POLR2, POLRA, RPB1, RPBh1, RPO2, RPOL2, RpIILS, hRPB220, hsRPB1	NP_000928.1	RNAP_II_Rpb1	57	1	0	QuickGO	Found
23067	SETD1B	SET domain containing 1B, histone lysine methyltransferase	KMT2G; Set1B	NP_001340274.1	RRM	63	3	4	RBPDB	Found
56252	YLPM1	YLP motif containing 1	ZAP3; ZAP113; PPP1R169; C14orf170	NP_062535.2	RRM	59	2	0	QuickGO	Found
155435	RBM33	RNA binding motif protein 33	PRR8	NP_444271.2	RRM	40	3	1	RBPDB/QuickGO	Found
23082	PPRC1	PPARG related coactivator 1	PRC	NP_055877.3	RRM	38	2	1	RBPDB/AttRACT/QuickGO	Found
9739	SETD1A	SET domain containing 1A, histone lysine methyltransferase	Set1; KMT2F; Set1A	NP_055527.1	RRM	33	3	4	RBPDB	Found
57466	SCAF4	SR-related CTD associated factor 4	SRA4; SFRS15	NP_065757.1	RRM	30	1	1	QuickGO	Found
23112	TNRC6B	trinucleotide repeat containing adaptor 6B		NP_001155973.1	RRM	21	1	0	QuickGO	Found
4288	MKI67	marker of proliferation Ki-67	KIA; MIB-; MIB-1; PPP1R105	NP_002408.3	RRM	20	5	1	QuickGO	Found

11052	CPSF6	cleavage and polyadenylation specific factor 6	CFIM; CFIM68; CFIM72; HPBRII-4; HPBRII-7	NP_001287876.1	RRM	19	0	0	RBPDB/QuickGO	Found
133522	PPARGC1B	PPARG coactivator 1 beta	PERC; ERRL1; PGC1B; PGC-1(beta)	NP_573570.3	RRM	14	2	1	RBPDB	Found
79171	RBM42	RNA binding motif protein 42		NP_077297.2	RRM	14	1	0	RBPDB/AttRACT/QuickGO	Found
8888	MCM3AP	minichromosome maintenance complex component 3 associated protein	GANP; SAC3; MAP80; PNRIID	NP_003897.2	RRM	12	1	0	RBPDB	Found
51593	SRRT	serrate, RNA effector molecule	ARS2; ASR2; serrate	NP_056992.4	RRM	12	0	1	RBPDB/QuickGO	Found
22985	ACIN1	apoptotic chromatin condensation inducer 1	ACN; ACINUS; fSAP152	NP_055792.1	RRM	11	1	0	RBPDB/QuickGO	Found
27327	TNRC6A	trinucleotide repeat containing adaptor 6A	GW1; FAME6; GW182; TNRC6; CAGH26	NP_055309.2	RRM	10	0	0	RBPDB/QuickGO	Found
23283	CSTF2T	cleavage stimulation factor subunit 2 tau variant	CstF-64T	NP_056050.1	RRM	9	0	0	RBPDB/QuickGO	Found
84875	PARP10	poly(ADP-ribose) polymerase family member 10	ARTD10	NP_116178.2	RRM	8	1	1	RBPDB	Found
23592	LEMD3	LEM domain containing 3	MAN1	NP_055134.2	RRM	6	0	0	RBPDB	Found
79869	CPSF7	cleavage and polyadenylation specific factor 7	CFIm59	NP_079087.3	RRM	5	0	1	RBPDB/QuickGO	Found
11030	RBPMS	RNA binding protein, mRNA processing factor	HERMES	NP_001008710.1	RRM	5	1	0	RBPDB/QuickGO	Found
9667	SAFB2	scaffold attachment factor B2		NP_055464.1	RRM	5	2	0	RBPDB/QuickGO	Found
8867	SYNJ1	synaptojanin 1	EIEE53; INPP5G; PARK20	NP_003886.3	RRM	19	2	7	RBPDB	Not found
57055	DAZ2	deleted in azoospermia 2	pDP1678	NP_065096.2	RRM	18	0	0	RBPDB	Not found
57054	DAZ3	deleted in azoospermia 3	pDP1679	NP_065097.2	RRM	15	0	0	RBPDB	Not found
57135	DAZ4	deleted in azoospermia 4	pDP1680; pDP1681	NP_001005375.1	RRM	14	0	0	RBPDB	Not found
8871	SYNJ2	synaptojanin 2	INPP5H	NP_003889.1	RRM	12	3	3	RBPDB	Not found
1975	EIF4B	eukaryotic translation initiation factor 4B	EIF-4B; PRO1843	NP_001287750.1	RRM	8	1	1	RBPDB/AttRACT/QuickGO	Not found

10495	ENOX2	ecto-NOX disulfide-thiol exchanger 2	APK1; tNOX; COVA1	NP_872114.1	RRM	6	1	0	RBPDB	Not found
54625	PARP14	poly(ADP-ribose) polymerase family member 14	BAL2; ARTD8; pART8; PARP-14	NP_060024.2	RRM	6	1	1	RBPDB	Not found
27037	TRMT2A	tRNA methyltransferase 2 homolog A	HTF9C	NP_073564.3	RRM	6	0	0	RBPDB/QuickGO	Not found
9908	G3BP2	G3BP stress granule assembly factor 2		NP_987101.1	RRM	5	1	1	RBPDB/AttRACT/QuickGO	Not found
22828	SCAF8	SR-related CTD associated factor 8	RBM16	NP_001273117.1	RRM; CID	24	1	0	RBPDB	Found
84456	L3MBTL3	L3MBTL histone methyl-lysine binding protein 3	MBT1; MBT-1	NP_115814.1	RRM; La	8	0	1	RBPDB	Found
58517	RBM25	RNA binding motif protein 25	S164; NET52; RNPC7; Snu71; RED120; fSAP94	NP_067062.1	RRM; PWI	8	1	0	RBPDB/AttRACT/QuickGO	Found
10594	PRPF8	pre-mRNA processing factor 8	PRP8; RP13; HPRP8; PRPC8; SNRNP220	NP_006436.3	RRM; U5-snRNA; U6-snRNA; RNaseH-like	9	0	0	QuickGO	Found
282996	RBM20	RNA binding motif protein 20		NP_001127835.2	RRM; Znf_C2H2x2	14	1	2	RBPDB	Found
54439	RBM27	RNA binding motif protein 27	Psc1; ARRS1; ZC3H18; ZC3H20	NP_061862.1	RRM; Znf_CCCH	12	1	0	RBPDB/QuickGO	Found
64852	TUT1	terminal uridylyl transferase 1, U6 snRNA-specific	PAPD2; RBM21; TENT1; URLC6; TUTase; STARPAP	NP_073741.3	RRM; Znf_CCCH	10	1	0	RBPDB/AttRACT/QuickGO	Found
55696	RBM22	RNA binding motif protein 22	Cwc2; ZC3H16; fSAP47	NP_060517.1	RRM; Znf_CCHC; Znf_CCCH	7	0	0	RBPDB/QuickGO	Found
2130	EWSR1	EWS RNA binding protein 1	EWS; EWS-FLI1; bK984G1.4	NP_053733.2	RRM; Znf_RanBP2	13	2	0	RBPDB/QuickGO	Found
4850	CNOT4	CCR4-NOT transcription complex subunit 4	NOT4; NOT4H; CLONE243	NP_001177779.1	RRM; Znf_RING	5	0	0	RBPDB/AttRACT/QuickGO	Not found
57690	TNRC6C	Trinucleotide repeat-containing gene 6C protein		NP_001136112.1	RRMx1	20	0	0	RBPDB/QuickGO	Found
146713	RBFOX3	RNA binding fox-1 homolog 3	FOX3; NEUN; FOX-3; HRNBP3	NP_001076044	RRMx1	6	1	0	RBPDB/QuickGO	Found
6421	SFPQ	splicing factor proline and glutamine rich	PSF; POMP100; PPP1R140	NP_005057.1	RRMx2	20	0	1	RBPDB/AttRACT/QuickGO	Found
10262	SF3B4	splicing factor 3b subunit 4	AFD1; Hsh49; SAP49; SF3b49	NP_005841.1	RRMx2	17	2	2	RBPDB/QuickGO	Found

22849	CPEB3	cytoplasmic polyadenylation element binding protein 3		NP_055727.3	RRMx2	13	1	0	RBPDB/QuickGO	Found
26528	DAZAP1	DAZ associated protein 1		NP_733829.1	RRMx2	9	1	0	RBPDB/AttRACT/Quick GO	Found
132864	CPEB2	cytoplasmic polyadenylation element binding protein 2	CPEB-2; CPE-BP2; hCPEB-2	NP_001170853.1	RRMx2	8	1	0	RBPDB/AttRACT/Quick GO	Found
55599	RNPC3	RNA binding region (RNP1, RRM) containing 3	RNP; IGHD5; RBM40; SNRNP65	NP_060089.1	RRMx2	8	0	0	RBPDB	Found
10180	RBM6	RNA binding motif protein 6	3G2; g16; DEF3; DEF-3; HLC-11; NY-LU-12	NP_005768.1	RRMx2	7	1	1	RBPDB/AttRACT/Quick GO	Found
64506	CPEB1	cytoplasmic polyadenylation element binding protein 1	CPEB; CPEB-1; h-CPEB; CPE-BP1; hCPEB-1	NP_085097.3	RRMx2	6	1	0	RBPDB/AttRACT	Found
3191	HNRNPL	heterogeneous nuclear ribonucleoprotein L	HNRPL; hnRNP-L; P/OKcl.14	NP_001524.2	RRMx2	6	0	0	RBPDB/AttRACT/Quick GO	Found
55269	PSPC1	paraspeckle component 1	PSP1	NP_001035879.1	RRMx2	6	0	0	RBPDB/QuickGO	Found
80315	CPEB4	cytoplasmic polyadenylation element binding protein 4	CPE-BP4; hCPEB-4	NP_085130.2	RRMx2	5	1	0	RBPDB/AttRACT/Quick GO	Found
9665	MARF1	meiosis regulator and mRNA stability factor 1	LKAP; LMKB; PPP1R34; KIAA0430	NP_055462.2	RRMx2	9	0	0	RBPDB	Not found
27303	RBMS3	RNA binding motif single stranded interacting protein 3		NP_001003793.1	RRMx2	5	0	0	RBPDB/AttRACT/Quick GO	Not found
8241	RBM10	RNA binding motif protein 10	S1-1; TARPS; GPATC9; ZRANB5; GPATCH9; DXS8237E	NP_005667.2	RRMx2; Znf_C2H2; Znf_RanBP2	5	1	1	RBPDB/QuickGO	Found
64062	RBM26	RNA binding motif protein 26	ARRS2; SE70-2; ZC3H17; PRO1777; C13orf10; PPP1R132	NP_001273560.1	RRMx2; Znf_CCCH	14	0	0	RBPDB/QuickGO	Found
1617	DAZ1	deleted in azoospermia 1	DAZ; SPGY	NP_004072.3	RRMx3	16	0	0	RBPDB	Found
125950	RAVER1	ribonucleoprotein, PTB binding 1		NP_001353103.1	RRMx3	11	0	2	RBPDB/QuickGO	Found
80004	ESRP2	epithelial splicing regulatory protein 2	RBM35B	NP_001352193.1	RRMx3	8	0	0	RBPDB/AttRACT/Quick GO	Found

54845	ESRP1	epithelial splicing regulatory protein 1	RBM35A; RMB35A; DFNB109	NP_060167.2	RRMx3	6	0	0	RBPDB/AttRACT/Quick GO	Found
22827	PUF60	poly(U) binding splicing factor 60	FIR; VRJS; RoBPI; SIAHBP1	NP_510965.1	RRMx3	6	0	0	RBPDB/QuickGO	Found
29890	RBM15B	RNA binding motif protein 15B	OTT3; HsOTT3; HUMAGCGB	NP_037418.3	RRMx3	6	1	0	RBPDB/QuickGO	Found
11189	CELF3	CUGBP Elav-like family member 3	CAGH4; ERDA4; ETR-1; TNRC4; BRUNOL1	NP_009116.3	RRMx3	5	0	0	QuickGO	Found
56853	CELF4	CUGBP Elav-like family member 4	CELF-4; BRUNOL4	NP_00134066 9.1	RRMx3	5	0	0	AttRACT/QuickGO	Found
60680	CELF5	CUGBP Elav-like family member 5	CELF-5; BRUNOL5; BRUNOL-5	NP_068757.2	RRMx3	5	0	0	AttRACT	Found
16686 3	RBM46	RNA binding motif protein 46	CT68	NP_659416.1	RRMx3	5	0	1	RBPDB/AttRACT	Not found
23013	SPEN	spen family transcriptional repressor	MINT; SHARP; RBM15C; HIAA0929	NP_055816.2	RRMx4	37	0	1	RBPDB/QuickGO	Found
10137	RBM12	RNA binding motif protein 12	SWAN; SCZD19; HRIHFB2091	NP_006038.2	RRMx4	18	0	0	RBPDB/QuickGO	Found
80336	PABPC1L	poly(A) binding protein cytoplasmic 1 like	EPAB; PABPC1L1; C20orf119; dJ1069P2.3	NP_00111822 8.1	RRMx4	6	0	1	RBPDB	Not found
60677	CELF6	CUGBP Elav-like family member 6	BRUNOL6	NP_443072.3	RRMx5	6	0	0	AttRACT	Not found
9904	RBM19	RNA binding motif protein 19	Mrd1	NP_00114017 1.1	RRMx6	5	0	0	RBPDB/QuickGO	Found
57805	CCAR2	cell cycle and apoptosis regulator 2	DBC1; DBC-1; NET35; p30DBC; p30 DBC; KIAA1967	NP_066997.3	S1	7	2	1	QuickGO	Found
1659	DHX8	DEAH-box helicase 8	DDX8; Dhr2; HRH1; PRP22; PRPF22	NP_004932.1	S1	7	1	0	RBPDB/QuickGO	Found
6830	SUPT6H	SPT6 homolog, histone chaperone and transcription elongation factor	SPT6; SPT6H; emb-5	NP_00130768 4.1	S1	5	1	0	RBPDB/QuickGO	Found
55095	SAMD4B	sterile alpha motif domain containing 4B	SMGB; Smaug2	NP_060498.2	SAM	7	1	0	RBPDB/QuickGO	Not found
20119 1	SAMD14	sterile alpha motif domain containing 14		NP_777580.1	SAM	6	0	0	RBPDB	Not found

10992	SF3B2	splicing factor 3b subunit 2	Cus1; SF3b1; SAP145; SF3B145; SF3b150	NP_006833.2	SAP	17	1	1	QuickGO	Found
11100	HNRNPUL1	heterogeneous nuclear ribonucleoprotein U like 1	E1BAP5; E1B-AP5; HNRPUL1	NP_008971.2	SAP; SPRY	12	0	0	QuickGO	Found
3192	HNRNPU	heterogeneous nuclear ribonucleoprotein U	SAFA; HNRPU; SAF-A; U21.1; pp120; EIEE54; GRIP120; hnRNP U; HNRNPU-AS1	NP_114032.2	SAP; SPRY	7	0	1	AttRACT/QuickGO	Found
10523	CHERP	calcium homeostasis endoplasmic reticulum protein	SRA1; DAN16; SCAF6	NP_006378.3	SURP	22	1	1	RBPDB/QuickGO	Not found
10291	SF3A1	splicing factor 3a subunit 1	PRP21; PRPF21; SAP114; SF3A120	NP_005868.1	SURPx2	21	0	0	RBPDB/QuickGO	Found
23518	R3HDM1	R3H domain containing 1	R3HDM	NP_001269727.1	SUZ	14	0	0	QuickGO	Found
7011	TEP1	telomerase associated protein 1	TP1; TLP1; p240; TROVE1; VAULT2	NP_009041.2	TROVE	12	1	4	RBPDB	Found
25929	GEMIN5	gem nuclear organelle associated protein 5	GEMIN-5	NP_056280.2	WD40_repeat	9	2	0	QuickGO	Found
64848	YTHDC2	YTH domain containing 2	CAHL; hYTHDC2	NP_073739.3	YTH	8	0	1	RBPDB/QuickGO	Found
51441	YTHDF2	YTH N6-methyladenosine RNA binding protein 2	CAHL; HGRG8; NY-REN-2	NP_057342.2	YTH	5	0	0	RBPDB/QuickGO	Found
253943	YTHDF3	YTH N6-methyladenosine RNA binding protein 3		NP_689971.4	YTH	5	0	0	RBPDB/QuickGO	Found
54915	YTHDF1	YTH N6-methyladenosine RNA binding protein 1	C20orf21	NP_060268.2	YTH	7	0	0	RBPDB/QuickGO	Not found
23131	GPATCH8	G-patch domain containing 8	GPATC8; KIAA0553	NP_001002909.1	zf-C2H2	14	0	1	QuickGO	Found
8175	SF3A2	splicing factor 3a subunit 2	PRP11; SAP62; PRPF11; SF3a66	NP_009096.2	Znf_C2H2	32	0	0	QuickGO	Found
8470	SORBS2	sorbin and SH3 domain containing 2	ARGBP2; PRO0618	NP_001257700.1	Znf_C2H2	14	0	3	QuickGO	Found
6631	SNRPC	small nuclear ribonucleoprotein polypeptide C	U1C; Yhc1	NP_003084.1	Znf_C2H2	9	0	0	RBPDB/QuickGO	Found
90850	ZNF598	zinc finger protein 598	HEL2	NP_835461.2	Znf_C2H2	11	1	1	QuickGO	Not found
85441	HELZ2	helicase with zinc finger 2	PDIP-1; PRIC285	NP_001032412.2	Znf_C2H2	9	0	1	QuickGO	Not found
79724	ZNF768	zinc finger protein 768		NP_078947.3	Znf_C2H2x10	10	2	0	QuickGO	Found

29803	REPIN1	replication initiator 1	AP4; RIP60; ZNF464; Zfp464	NP_037532.2	Znf_C2H2x15	7	1	0	QuickGO	Found
7756	ZNF207	zinc finger protein 207	BuGZ; hBuGZ	NP_001091977.1	Znf_C2H2x2	21	0	0	QuickGO	Found
27332	ZNF638	zinc finger protein 638	ZFML; NP220; Zfp638	NP_001014972.1	Znf_C2H2x2; RRMx2	8	1	0	RBPDB/AttRACT/QuickGO	Found
23217	ZFR2	zinc finger RNA binding protein 2	KIAA1086	NP_055989.1	Znf_C2H2x3	20	0	1	RBPDB	Found
25946	ZNF385A	zinc finger protein 385A	HZF; RZF; ZFP385; ZNF385	NP_001124439.1	Znf_C2H2x3	8	2	0	QuickGO	Found
163033	ZNF579	zinc finger protein 579		NP_689813.2	Znf_C2H2x8	12	0	3	QuickGO	Found
5514	PPP1R10	protein phosphatase 1 regulatory subunit 10	p99; FB19; R111; CAT53; PNUTS; PP1R10	NP_002705.2	Znf_CCCH	17	0	3	RBPDB/QuickGO	Found
124245	ZC3H18	zinc finger CCCH-type containing 18	NHN1	NP_001281269.1	Znf_CCCH	16	0	1	RBPDB/QuickGO	Found
23144	ZC3H3	zinc finger CCCH-type containing 3	ZC3HDC3	NP_055932.2	Znf_CCCH	14	0	2	RBPDB	Found
196441	ZFC3H1	zinc finger C3H1-type containing	CSRC2; PSRC2; CCDC131	NP_659419.3	Znf_CCCH	13	0	1	QuickGO	Found
80149	ZC3H12A	zinc finger CCCH-type containing 12A	Reg1; MCPIP; MCPIP1; MCPIP-1; dJ423B22.1	NP_001310479.1	Znf_CCCH	12	0	2	RBPDB/QuickGO	Found
340152	ZC3H12D	zinc finger CCCH-type containing 12D	TFL; p34; MCPIP4; C6orf95; dJ281H8.1	NP_997243.2	Znf_CCCH	10	2	0	RBPDB	Found
23091	ZC3H13	zinc finger CCCH-type containing 13	Xio; KIAA0853	NP_001070256.1	Znf_CCCH	9	1	1	RBPDB/QuickGO	Found
9931	HELZ	helicase with zinc finger	DHRC; DRHC; HUMORF5	NP_001317376.1	Znf_CCCH	21	1	4	RBPDB/QuickGO	Not found
85463	ZC3H12C	zinc finger CCCH-type containing 12C	MCPIP3	NP_203748.1	Znf_CCCH	7	1	2	RBPDB	Not found
94059	LENG9	leukocyte receptor cluster member 9		NP_945339.2	Znf_CCCH	6	0	1	RBPDB	Not found
80742	PRR3	proline rich 3	CAT56	NP_079539.2	Znf_CCCH	6	0	1	RBPDB/QuickGO	Not found
55621	TRMT1	tRNA methyltransferase 1	TRM1; MRT68	NP_060192.1	Znf_CCCH	6	0	0	RBPDB/QuickGO	Not found
340554	ZC3H12B	zinc finger CCCH-type containing 12B	MCPIP2; CXorf32	NP_001010888.3	Znf_CCCH	6	1	0	RBPDB	Not found
149041	RC3H1	ring finger and CCCH-type domains 1	RNF198; ROQUIN	NP_001287779.1	Znf_CCCH; Znf_RING	19	4	0	RBPDB/AttRACT/QuickGO	Not found

54542	RC3H2	ring finger and CCCH-type domains 2	MNAB; RNF164	NP_001094058.1	Znf_CCCH; Znf_RING	13	1	0	RBPDB/QuickGO	Not found
23211	ZC3H4	zinc finger CCCH-type containing 4	C19orf7	NP_055983.1	Znf_CCCHx2	39	1	1	RBPDB/QuickGO	Found
376940	ZC3H6	zinc finger CCCH-type containing 6	ZC3HDC6	NP_940983.2	Znf_CCCHx2	14	0	0	RBPDB	Found
7538	ZFP36	ZFP36 ring finger protein	TTP; G0S24; GOS24; TIS11; NUP475; zfp-36; RNF162A	NP_003398.3	Znf_CCCHx2	10	1	0	RBPDB/AttRACT/QuickGO	Found
79882	ZC3H14	zinc finger CCCH-type containing 14	SUT2; MRT56; UKp68; MSUT-2; NY-REN-37	NP_079100.2	Znf_CCCHx2	8	0	1	RBPDB/QuickGO	Found
678	ZFP36L2	ZFP36 ring finger protein like 2	BRF2; ERF2; ERF-2; TIS11D; RNF162C	NP_008818.3	Znf_CCCHx2	9	0	0	RBPDB/AttRACT/QuickGO	Not found
23318	TUT4	terminal uridylyl transferase 4	PAPD3; TENT3A; ZCCHC11	NP_001009881.1	Znf_CCCHx3	15	3	0	QuickGO	Found
64718	UNKL	unk like zinc finger	ZC3H5L; C16orf28; ZC3HDC5L	NP_001180317.2	Znf_CCCHx3	8	1	1	RBPDB	Not found
84872	ZC3H10	zinc finger CCCH-type containing 10	ZC3HDC10	NP_116175.1	Znf_CCCHx3	5	0	0	RBPDB/AttRACT/QuickGO	Not found
7681	MKRN3	makorin ring finger protein 3	CPPB2; D15S9; RNF63; ZFP127; ZNF127	NP_005655.1	Znf_CCCHx3; Znf_RING	6	0	1	RBPDB	Not found
4154	MBNL1	muscleblind like splicing regulator 1	EXP; MBNL	NP_066368.2	Znf_CCCHx4	6	0	1	RBPDB/AttRACT/QuickGO	Found
10150	MBNL2	muscleblind like splicing regulator 2	MBLL; MBLL39; PRO2032	NP_659002.1	Znf_CCCHx4	5	0	0	RBPDB/QuickGO	Found
55796	MBNL3	muscleblind like splicing regulator 3	CHCR; MBLX; MBXL; MBLX39	NP_060858.2	Znf_CCCHx4	6	0	0	RBPDB/QuickGO	Not found
23264	ZC3H7B	zinc finger CCCH-type containing 7B	RoXaN; ROXAN1	NP_060060.3	Znf_CCCHx5	9	0	0	RBPDB/QuickGO	Not found
85451	UNK	unk zinc finger	ZC3H5; UNKEMPT; ZC3HDC5	NP_001073888.2	Znf_CCCHx5	8	0	0	RBPDB/QuickGO	Not found
5930	RBBP6	RB binding protein 6, ubiquitin ligase	PACT; MY038; P2P-R; RBQ-1; SNAMA	NP_008841.2	Znf_CCHC	13	0	2	QuickGO	Found
23089	PEG10	paternally expressed 10	EDR; HB-1; Mar2; RTL2; MEF3L; Mart2; RGAG3; SIRH1	NP_001165908.1	Znf_CCHC	12	0	1	QuickGO	Found
55596	ZCCHC8	zinc finger CCHC-type containing 8		NP_060082.2	Znf_CCHC	6	0	0	QuickGO	Found

79670	TUT7	terminal uridylyl transferase 7	PAPD6; TENT3B; ZCCHC6	NP_078893.2	Znf_CCHCx3	7	3	0	QuickGO	Found
84186	ZCCHC7	zinc finger CCHC-type containing 7	AIR1; HSPC086	NP_001276050.1	Znf_CCHCx4	5	0	1	QuickGO	Found
9169	SCAF11	SR-related CTD associated factor 11	SIP1; CASP11; SFRS2IP; SRRP129; SRSF2IP	NP_004710.2	Znf_RING	5	0	0	QuickGO	Found
9666	DZIP3	DAZ interacting zinc finger protein 3	UURF2; PPP1R66; hRUL138	NP_055463.1	Znf_RING	6	0	1	QuickGO	Not found
131405	TRIM71	tripartite motif containing 71	LIN41; LIN-41	NP_001034200.1	Znf_RING	5	1	1	QuickGO	Not found
10270	AKAP8	A-kinase anchoring protein 8	AKAP-8; AKAP95; AKAP 95; AKAP-95	NP_005849.1	Znf-C2H2	5	1	0	QuickGO	Found
58508	KMT2C	lysine methyltransferase 2C	HALR; MLL3; KLEFS2	NP_733751.2	Znf-RanBP2	62	2	3	QuickGO	Found
7916	PRRC2A	proline rich coiled-coil 2A	G2; BAT2; D6S51; D6S51E	NP_004629.3		68	5	6	QuickGO	Found
23524	SRRM2	serine/arginine repetitive matrix 2	CWF21; Cwc21; 300-KD; SRL300; SRm300; HSPC075	NP_057417.3		32	2	4	RBPDB/QuickGO	Found
2316	FLNA	filamin A	FLN; FMD; MNS; OPD; ABPX; CSBS; CVD1; FGS2; FLN1; NHBP; OPD1; OPD2; XLVD; XMVD; FLN-A; ABP-280	NP_001104026.1		31	0	5	QuickGO	Found
57666	FBRSL1	fibrosin like 1		NP_001136113.1		27	4	3	QuickGO	Found
51729	WBP11	WW domain binding protein 11	NPWBP; SIPP1; WBP-11; PPP1R165	NP_057396.1		27	3	2	QuickGO	Found
27043	PELP1	proline, glutamate and leucine rich protein 1	MNAR; P160	NP_055204.4		26	3	0	QuickGO	Found
11083	DIDO1	death inducer-obliterator 1	BYE1; DIO1; DATF1; DIDO2; DIDO3; DIO-1; DATF-1; C20orf158; dJ885L7.8	NP_149072.2		25	0	0	QuickGO	Found
11273	ATXN2L	ataxin 2 like	A2D; A2LG; A2LP; A2RP	NP_009176.2		24	0	0	QuickGO	Found
55339	WDR33	WD repeat domain 33	NET14; WDC146	NP_060853.3		22	2	0	QuickGO	Found

1981	EIF4G1	eukaryotic translation initiation factor 4 gamma 1	P220; EIF4F; EIF4G; EIF4GI; PARK18; EIF-4G1	NP_886553.3		21	2	1	QuickGO	Found
1107	CHD3	chromodomain helicase DNA binding protein 3	ZFH; Mi-2a; SNIBCPS; Mi2-ALPHA	NP_001005273.1		18	0	1	QuickGO	Found
54954	FAM120C	family with sequence similarity 120C	ORF34; CXorf17	NP_060318.4		18	0	0	QuickGO	Found
10075	HUWE1	HECT, UBA and WWE domain containing E3 ubiquitin protein ligase 1	MULE; Ib772; LASU1; MRXST; UREB1; HECTH9; URE-B1; ARF-BP1; HSPC272	NP_113584.3		18	0	0	QuickGO	Found
7791	ZYX	zyxin	ESP-2; HED-2	NP_003452.1		18	0	0	QuickGO	Found
8621	CDK13	cyclin dependent kinase 13	CHED; CDC2L; CDC2L5; hCDK13; CHDFIDD	NP_003709.3		17	1	0	QuickGO	Found
10915	TCERG1	transcription elongation regulator 1	Urn1; CA150; TAF2S	NP_006697.2		17	2	1	QuickGO	Found
6942	TCF20	transcription factor 20	AR1; SPBP; TCF-20	NP_005641.1		17	0	2	QuickGO	Found
57142	RTN4	reticulon 4	ASY; NSP; NOGO; RTN-X; NSP-CL; RTN4-A; RTN4-C; RTN4-B1; RTN4-B2; NI220/250; Nbla00271; Nbla10545	NP_065393.1		15	1	2	QuickGO	Found
311	ANXA11	annexin A11	ALS23; AN1; CAP50; CAP-50	NP_001148.1		13	0	0	QuickGO	Found
79080	CCDC86	coiled-coil domain containing 86		NP_077003.1		12	0	0	QuickGO	Found
25962	VIRMA	vir like m6A methyltransferase associated	MSTP054; fSAP121; KIAA1429	NP_056311.2		12	3	3	QuickGO	Found
9530	BAG4	BCL2 associated athanogene 4	SODD; BAG-4	NP_004865.1		11	1	1	QuickGO	Found
1759	DNM1	dynamin 1	DNM; EIEE31	NP_004399.2		11	2	4	QuickGO	Found
4204	MECP2	methyl-CpG binding protein 2	RS; RTS; RTT; PPMX; MR6; MRX79; MRXSL; AUTSX3; MRXS13	NP_004983.1		11	0	0	QuickGO	Found

5591	PRKDC	protein kinase, DNA-activated, catalytic subunit	HYRC; p350; DNAPK; DNPK1; HYRC1; IMD26; XRCC7; DNAPKc; DNA-PKC; DNA-PKcs	NP_008835.5		11	0	0	QuickGO	Found
9898	UBAP2L	ubiquitin associated protein 2 like	NICE4; NICE-4	NP_055662.3		11	0	0	QuickGO	Found
27340	UTP20	UTP20 small subunit processome component	DRIM; 1A6/DRIM	NP_055318.2		11	0	1	QuickGO	Found
6829	SUPT5H	SPT5 homolog, DSIF elongation factor subunit	SPT5; SPT5H; Tat-CT1	NP_003160.2		10	0	1	QuickGO	Found
118	ADD1	adducin 1	ADDA	NP_001341690.1		9	0	0	QuickGO	Found
23246	BOP1	BOP1 ribosomal biogenesis factor		NP_056016.1		9	0	1	QuickGO	Found
81608	FIP1L1	factor interacting with PAPOLA and CPSF1	Rhe; FIP1; hFip1	NP_112179.2		9	0	0	QuickGO	Found
10514	MYBBP1A	MYB binding protein 1a	P160; PAP2; Pol5	NP_001099008.1		9	0	1	QuickGO	Found
22864	R3HDM2	R3H domain containing 2	CAG6; PR01365	NP_001317050.1		9	0	0	QuickGO	Found
23076	RRP1B	ribosomal RNA processing 1B	Nnp1; RRP1; NNP1L; KIAA0179; PPP1R136	NP_055871.1		9	0	1	QuickGO	Found
6949	TCOF1	treacle ribosome biogenesis factor 1	TCS; MFD1; TCS1; treacle	NP_001128715.1		9	0	0	QuickGO	Found
7015	TERT	telomerase reverse transcriptase	TP2; TRT; CMM9; EST2; TCS1; hTRT; DKCA2; DKCB4; hEST2; PFBMFT1NP_937983.2	NP_937983.2		9	2	3	QuickGO	Found
63893	UBE2O	ubiquitin conjugating enzyme E2 O	E2-230K	NP_071349.3		9	0	1	QuickGO	Found
57602	USP36	ubiquitin specific peptidase 36	DUB1	NP_001308220.1		9	1	0	QuickGO	Found
904	CCNT1	cyclin T1	CCNT; CYCT1; HIVE1	NP_001231.2		8	0	0	QuickGO	Found
4150	MAZ	MYC associated zinc finger protein	PUR1; ZF87; Pur-1; SAF-1; SAF-2; SAF-3; Zif87; ZNF801	NP_001036004.1		8	0	0	QuickGO	Found

310	ANXA7	annexin A7	SNX; ANX7; SYNEXIN	NP_004025.1		7	0	0	QuickGO	Found
984	CDK11B	cyclin dependent kinase 11B	p58; PK58; CDK11; CLK-1; CDC2L1; PITSLREA; p58CLK-1; CDK11-p46; CDK11-p58; p58CDC2L1; CDK11-p110	NP_001778.2		7	1	1	QuickGO	Found
23019	CNOT1	CCR4-NOT transcription complex subunit 1	NOT1; CDC39; NOT1H; AD-005	NP_057368.3		7	1	0	QuickGO	Found
5394	EXOSC10	exosome component 10	p2; p3; p4; RRP6; PMSCL; Rrp6p; PM-Scl; PMSCL2; PM/Scl-100	NP_001001998.1		7	0	0	QuickGO	Found
3428	IFI16	interferon gamma inducible protein 16	PYHIN2; IFNGIP1	NP_001351796.1		7	0	0	QuickGO	Found
23028	KDM1A	lysine demethylase 1A	AOF2; CPRF; KDM1; LSD1; BHC110	NP_001009999.1		7	0	0	QuickGO	Found
56257	MEPCE	methylphosphate capping enzyme	BCDIN3	NP_062552.2		7	0	1	QuickGO	Found
4928	NUP98	nucleoporin 9	ADIR2; NUP96; NUP196	NP_001352055.1		7	1	0	QuickGO	Found
5586	PKN2	protein kinase N2	PAK2; PRK2; STK7; Pak-2; PRKCL2; PRO2042	NP_006247.1		7	0	1	QuickGO	Found
6733	SRPK2	SRSF protein kinase 2	SFRSK2	NP_001337669.1		7	0	0	QuickGO	Found
23272	TASOR	transcription activation suppressor	RAP140; TASOR1; se89-1; C3orf63; FAM208A	NP_001352564.1		7	0	1	QuickGO	Found
7175	TPR	translocated promoter region, nuclear basket protein		NP_003283.2		7	4	1	QuickGO	Found
7343	UBTF	upstream binding transcription factor	UBF; UBF1; UBF2; UBF-1; CONDBA; NOR-90	NP_055048.1		7	0	1	QuickGO	Found
8531	YBX3	Y-box binding protein 3	CSDA; DBPA; CSDA1; ZONAB	NP_003642.3		7	1	0	QuickGO	Found
1106	CHD2	chromodomain helicase DNA binding protein 2	EEOC	NP_001262.3		6	0	0	QuickGO	Found

79066	METTL16	methyltransferase like 16	METT10D	NP_076991.3		6	2	1	QuickGO	Found
9221	NOLC1	nucleolar and coiled-body phosphoprotein 1	P130; NOPP130; NOPP140; NS5ATP13	NP_001271317.1		6	0	1	QuickGO	Found
25957	PNISR	PNN interacting serine and arginine rich protein	SFRS18; HSPC306; SRrp130; C6orf111; bA98I9.2	NP_001309334.1		6	0	1	QuickGO	Found
10155	TRIM28	tripartite motif containing 28	KAP1; TF1B; RNF96; TIF1B; PPP1R157	NP_005753.1		6	0	1	QuickGO	Found
23350	U2SURP	U2 snRNP associated SURP domain containing	SR140; fSAPa	NP_001073884.1		6	2	0	QuickGO	Found
9875	URB1	URB1 ribosome biogenesis homolog	NPA1; C21orf108	NP_055640.2		6	0	1	QuickGO	Found
23	ABCF1	ATP binding cassette subfamily F member 1	ABC27; ABC50	NP_001020262.1		5	0	0	QuickGO	Found
58509	CACTIN	cactin, spliceosome C complex subunit	fSAPc; C19orf29; NY-REN-24	NP_001074012.1		5	0	1	QuickGO	Found
9343	EFTUD2	elongation factor Tu GTP binding domain containing 2	MFDM; MFDGA; Snu114; Snrp116; SNRNP116; U5-116KD	NP_004238.3		5	0	0	QuickGO	Found
57727	NCOA5	nuclear receptor coactivator 5	CIA; bA465L10.6	NP_066018.1		5	0	0	QuickGO	Found
54888	NSUN2	NOP2/Sun RNA methyltransferase 2	MISU; MRT5; SAKI; TRM4	NP_060225.4		5	0	0	QuickGO	Found
10940	POP1	POP1 homolog, ribonuclease P/MRP subunit	ANXD2	NP_001139332.1		5	0	0	QuickGO	Found
51535	PPHLN1	periphilin 1	CR; HSPC206; HSPC232	NP_057572.5		5	0	0	QuickGO	Found
57109	REXO4	REX4 homolog, 3'-5' exonuclease	REX4; XPMC2; XPMC2H	NP_065118.2		5	0	0	QuickGO	Found
57794	SUGP1	SURP and G-patch domain containing 1	RBP; SF4; F23858	NP_757386.2		5	0	0	QuickGO	Found
81550	TDRD3	tudor domain containing 3		NP_001139542.1		5	2	0	QuickGO	Found
9100	USP10	ubiquitin specific peptidase 10	UBPO	NP_001259004.1		5	0	0	QuickGO	Found
134430	WDR36	WD repeat domain 36	GLC1G; UTP21; TAWDRP; TA-WDRP	NP_644810.1		5	0	0	QuickGO	Found

55135	WRAP53	WD repeat containing antisense to TP53	DKCB3; TCAB1; WDR79	NP_060551.2		5	0	1	QuickGO	Found
23215	PRRC2C	proline rich coiled-coil 2C	XTP2; BAT2D1; BAT2L2; BAT2-iso	NP_055987.2		34	2	3	QuickGO	Not found
2317	FLNB	filamin B	AOI; FH1; SCT; TAP; LRS1; TABP; FLN-B; FLN1L; ABP-278; ABP-280	NP_00115778 9.1		32	0	6	QuickGO	Not found
7373	COL14A1	collagen type XIV alpha 1 chain	UND	NP_066933.1		28	1	1	QuickGO	Not found
6311	ATXN2	ataxin 2	ATX2; SCA2; TNRC13	NP_002964.4		26	2	2	QuickGO	Not found
7145	TNS1	tensin 1	TNS; MXRA6; MST091; MST122; MST127; MSTP091; MSTP122; MSTP127; PPP1R155	NP_072174.3		25	0	3	QuickGO	Not found
1729	DIAPH1	diaphanous related formin 1	DIA1; DRF1; DFNA1; LFHL1; SCBMS; hDIA1	NP_005210.3		24	1	1	QuickGO	Not found
16590 4	XIRP1	xin actin binding repeat containing 1	Xin; CMYA1	NP_919269.2		22	1	0	QuickGO	Not found
4035	LRP1	LDL receptor related protein 1	APR; KPA; LRP; A2MR; CD91; APOER; LRP1A; TGFBR5; IGFBP3R; IGFBP-3R; IGFBP3R1	NP_002323.2		20	1	2	QuickGO	Not found
79868	ALG13	ALG13 UDP-N-acetylglucosaminyltransfe rase subunit	CDG1S; EIEE36; MDS031; TDRD13; CXorf45; GLT28D1; YGL047W	NP_00109339 2.1		19	0	1	QuickGO	Not found
8672	EIF4G3	eukaryotic translation initiation factor 4 gamma 3	eIF4G 3; eIF4GII; eIF-4G 3	NP_00118573 0.1		18	1	1	QuickGO	Not found
84726	PRRC2B	proline rich coiled-coil 2B	BAT2L; BAT2L1; LQFBS-1; KIAA0515	NP_037450.2		18	2	5	QuickGO	Not found
22999	RIMS1	regulating synaptic membrane exocytosis 1	RIM; RIM1; CORD7; RAB3IP2	NP_055804.2		18	3	3	QuickGO	Not found
23255	MTCL1	microtubule crosslinking factor 1	SOGA2; CCDC165; KIAA0802	NP_056025.2		17	2	0	QuickGO	Not found

22862	FNDC3A	fibronectin type III domain containing 3A	HUGO; FNDC3; bA203I16.1; bA203I16.5	NP_001073141.1		13	0	2	QuickGO	Not found
2194	FASN	fatty acid synthase	FAS; OA-519; SDR27	NP_004095.4		12	0	1	QuickGO	Not found
10749	KIF1C	kinesin family member 1C	SAX2; LTXS1; SATX2; SPAX2; SPG58	NP_006603.2		12	1	2	QuickGO	Not found
11196	SEC23IP	SEC23 interacting protein	P125; P125A; MSTP053	NP_009121.1		12	0	0	QuickGO	Not found
23196	FAM120A	family with sequence similarity 120A	OSSA; C9orf10; HBVPTPAP	NP_055427.2		11	1	1	QuickGO	Not found
1778	DYNC1H1	dynein cytoplasmic 1 heavy chain 1	p22; DHC1; DNCL; DYHC; HL-3; CMT2O; DHC1a; DNCH1; DNECL; Dnchc1; SMALED1	NP_001367.2		10	0	1	QuickGO	Not found
831	CAST	calpastatin	BS-17; PLACK	NP_001741.4		9	0	0	QuickGO	Not found
26093	CCDC9	coiled-coil domain containing 9		NP_056418.1		9	1	0	QuickGO	Not found
26058	GIGYF2	GRB10 interacting GYF protein 2	GYF2; PERQ2; PERQ3; PARK11; TNRC15	NP_001096617.1		9	1	0	QuickGO	Not found
4134	MAP4	microtubule associated protein 4		NP_002366.2		9	1	0	QuickGO	Not found
54726	OTUD4	OTU deubiquitinase 4	HIN1; DUBA6; HSHIN1	NP_001352986.1		9	1	0	QuickGO	Not found
5339	PLEC	plectin	HD1; PCN; EBS1; EBSO; PLTN; EBSMD; EBSND; EBSOG; EBSPA; PLEC1; LGMD2Q; PLEC1b; LGMDR17	NP_000436.2		9	0	2	QuickGO	Not found
7205	TRIP6	thyroid hormone receptor interactor 6	OIP1; OIP-1; ZRP-1; TRIP-6; TRIP6i2	NP_003293.2		9	1	0	QuickGO	Not found
255101	CFAP65	cilia and flagella associated protein 65	CCDC108	NP_919278.2		8	0	0	QuickGO	Not found
64778	FNDC3B	fibronectin type III domain containing 3B	FAD104; PRO4979; YVTM2421	NP_073600.3		8	1	2	QuickGO	Not found
23499	MACF1	microtubule actin crosslinking factor 1	ACF7; LIS9; MACF; OFC4; ABP620	NP_036222.3		8	1	0	QuickGO	Not found

9138	ARHGEF1	Rho guanine nucleotide exchange factor 1	LSC; GEF1; LBCL2; SUB1.5; P115-RHOGEF	NP_945353.1		6	1	1	QuickGO	Not found
722	C4BPA	complement component 4 binding protein alpha	PRP; C4BP	NP_000706.1		6	0	0	QuickGO	Not found
10985	GCN1	GCN1 activator of EIF2AK4	GCN1L; GCN1L1; PRIC295	NP_006827.1		6	1	0	QuickGO	Not found
63897	HEATR6	HEAT repeat containing 6	ABC1	NP_071353.4		6	0	0	QuickGO	Not found
149986	LSM14B	FT005; LSM13; FAM61B; RAP55B; C20orf40; bA11M20.3	LSM family member 14B	NP_653304.2		6	0	1	QuickGO	Not found
84273	NOA1	nitric oxide associated 1	MTG3; hNOA1; C4orf14; hAtNOS1; mAtNOS1	NP_115689.1		6	1	0	QuickGO	Not found
9475	ROCK2	Rho associated coiled-coil containing protein kinase 2	ROCK-II	NP_004841.2		6	0	0	QuickGO	Not found
6238	RRBP1	ribosome binding protein 1	RRp; hES; ES130; ES/130	NP_001352542.1		6	0	0	QuickGO	Not found
79048	SECISBP2	SECIS binding protein 2	SBP2	NP_076982.3		6	0	0	QuickGO	Not found
11180	WDR6	WD repeat domain 6		NP_060501.4		6	0	3	QuickGO	Not found
51143	DYNC1LI1	dynein cytoplasmic 1 light intermediate chain 1	LIC1; DLC-A; DNCLI1	NP_057225.2		5	0	2	QuickGO	Not found
10539	GLRX3	glutaredoxin 3	GRX3; GRX4; GLRX4; PICOT; TXNL2; TXNL3	NP_001186797.1		5	0	0	QuickGO	Not found
2804	GOLGB1	golgin B1	GCP; GCP372; GOLIM1	NP_001243415.1		5	0	0	QuickGO	Not found
23708	GSPT2	G1 to S phase transition 2	GST2; ERF3B	NP_060564.2		5	1	1	QuickGO	Not found
4644	MYO5A	myosin VA	GS1; MYO5; MYH12; MYR12	NP_000250.3		5	0	0	QuickGO	Not found
553115	PEF1	penta-EF-hand domain containing 1	ABP32; PEF1A	NP_036524.1		5	0	0	QuickGO	Not found
55086	RADX	RPA1 related single stranded DNA binding protein, X-linked	CXorf57	NP_060485.4		5	0	1	QuickGO	Not found
81844	TRIM56	tripartite motif containing 56	RNF109	NP_112223.1		5	0	0	QuickGO	Not found
55833	UBAP2	ubiquitin associated protein 2	UBAP-2	NP_060919.3		5	0	0	QuickGO	Not found
